# Microplastics Generate Less Mineral Protection of Soil Carbon and More CO_2_ Emissions

**DOI:** 10.1002/advs.202409585

**Published:** 2024-12-30

**Authors:** Jia Shi, Andrew J. Tanentzap, Yuanze Sun, Jianjun Wang, Baoshan Xing, Matthias C. Rillig, Changchao Li, Ling Jin, Fang Wang, Tanveer M. Adyel, Jianying Shang, Xiang Wang, Jie Wang

**Affiliations:** ^1^ Key Laboratory of Arable Land Conservation (North China) College of Land Science and Technology China Agricultural University Beijing 100193 China; ^2^ Ecosystems and Global Change Group School of the Environment Trent University Peterborough K9L 0G2 Canada; ^3^ Beijing Key Laboratory of Farmland Soil Pollution Prevention and Remediation College of Resources and Environmental Sciences China Agricultural University Beijing 100193 China; ^4^ State Key Laboratory of Lake Science and Environment Nanjing Institute of Geography and Limnology Chinese Academic of Sciences Nanjing 210008 China; ^5^ Stockbridge School of Agriculture University of Massachusetts Amherst MA 01003 USA; ^6^ Institut für Biologie Freie Universität Berlin Altensteinstrasse 6 14195 Berlin Germany; ^7^ Department of Civil and Environmental Engineering The Hong Kong Polytechnic University Hung Hom Kowloon Hong Kong 999077 China; ^8^ Department of Health Technology and Informatics The Hong Kong Polytechnic University Hung Hom Kowloon Hong Kong 999077 China; ^9^ State Key Laboratory of Soil and Sustainable Agriculture Institute of Soil Science Chinese Academy of Sciences Nanjing 210008 China; ^10^ Bioscience and Food Technology Discipline RMIT University Melbourne VIC 3000 Australia

**Keywords:** CO_2_ emission, microplastic‐derived dissolved organic matter, microplastics, mineral‐associated organic carbon, minerals, natural organic matter, sorption

## Abstract

Microplastic pollution in terrestrial ecosystems threatens to destabilize large soil carbon stocks that help to mitigate climate change. Carbon‐based substrates can release from microplastics and contribute to terrestrial carbon pools, but how these emerging organic compounds influence carbon mineralization and sequestration remains unknown. Here, microcosm experiments are conducted to determine the bioavailability of microplastic‐derived dissolved organic matter (MP‐DOM) in soils and its contribution to mineral‐associated carbon pool. The underlying mechanisms are identified by estimating its spectroscopic and molecular signatures and comparing its sorption properties on model minerals with natural organic matter (NOM). The results show that MP‐DOM leads to 21–576% higher CO_2_ emissions and 34–83% lower mineral‐associated organic carbon in soils than NOM, depending on the type of plastic polymer. DOM from biodegradable microplastics induces higher CO_2_ emissions than conventional microplastics. It is found that MP‐DOM is 7.96 times more labile than NOM, making it more accessible for microbial utilization. The lower degree of humification, fewer polar functional groups, and higher H/C ratios in MP‐DOM also led to 3.96 times less sorption with mineral particles. The findings provide insights into the effects of microplastics on soil carbon storage and highlight their consequences for wider terrestrial carbon cycling and climate warming.

## Graphical abstract

This study experimentally determines the bioavailability of microplastic‐derived dissolved organic matter (MP‐DOM) in soils and its contribution to the soil mineral‐associated carbon pool. It is demonstatrated that MP‐DOM increase CO_2_ emissions and result in less mineral associated carbon storage than natural organic matter (NOM) because of its higher lability and lower sorption to minerals, potentially suggesting that MP‐DOM may reduce soil carbon storage capacity.

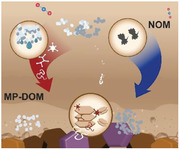

## 1 Introduction

Soil is the largest terrestrial organic carbon pool, stored in a large variety of organic molecules.^[^ ^1^, ^2^ ^]^ Release of this carbon as carbon dioxide (CO_2_) would exacerbate global warming, whereas increasing soil organic carbon can help mitigate climate change.^[^ ^3^ ^]^ Minerals are widely assumed to protect organic matter from degradation in the soil via chemical or physical association, thus promoting carbon sequestration.^[^ ^4^, ^5^ ^]^ However, organic molecules exhibit distinct mineral associations and persistence because of their diverse chemical traits (e.g., molecular weight, chemical structure, stoichiometry, oxidation state, and bioavailability).^[^ ^6^, ^7^, ^8^ ^]^ The capacity of organic molecules to associate with minerals and their bioavailability influence the long‐term trajectory of the soil carbon sink. Given that minor changes in soil carbon pool can dampen accelerated rate of CO_2_ emission and associated climate changes, understanding how anthropogenic environmental stressors, like plastic pollution, impact these interactions is critical for carbon cycling.

Plastic pollution is a global and increasing threat to many terrestrial ecosystems.^[^ ^9^, ^10^, ^11^, ^12^, ^13^ ^]^ As estimated, by 2050, ≈12000 million tons of plastic waste will end up in landfills or the natural environment.^[^ ^14^, ^15^ ^]^ This plastic waste partially fragments into microplastics (plastic particles <5 mm in diameter) due to environmental and biological activity.^[^ ^16^, ^17^ ^]^ Microplastics have various adverse effects on the abiotic and biotic components in soil ecosystems, including modifying physiochemical properties,^[^ ^18^ ^]^ interference with microbial functions,^[^ ^19^, ^20^ ^]^ affecting plant growth,^[^ ^21^, ^22^ ^]^ and threatening food web health.^[^ ^23^, ^24^ ^]^ In addition, dissolved organic matter (DOM) can originate from microplastics, termed herein microplastic‐derived DOM (MP‐DOM), via leaching and weathering processes. ^[^ ^25^, ^26^ ^]^ MP‐DOM may contain various labile and bioavailable plastic additives (e.g., plasticizers, colorants, and antioxidants) or carbon backbones (e.g., oligomers and/or monomers) derived from the plastic polymers themselves.^[^ ^27^, ^28^ ^]^ Considering the high proportion of carbon in microplastics, typically ≈80%, MP‐DOM may constitute an emerging source of soil organic carbon, and potentially alter the fate of soil carbon pools and microbiomes.^[^ ^29^, ^30^, ^31^ ^]^ Previous study demonstrated that microplastics altered soil chemodiversity and facilitated metabolisms with high carbon investment to decrease organic carbon storage.^[^ ^32^ ^]^ Despite its global importance, the environmental reactivity of MP‐DOM in soils has received insufficient attention, limiting our understanding of how microplastics impact soil carbon cycling.

The associations between MP‐DOM and soil minerals may differ from those of natural organic matter (NOM). Previous studies found that MP‐DOM was typically characterized by lower molecular weight, weaker aromaticity, and greater lability in comparison with dissolved NOM,^[^ ^28^, ^32^ ^]^ potentially indicating that MP‐DOM may be more readily utilized by soil microorganisms. Additionally, structural and compositional differences between molecules in MP‐DOM and NOM may cause different interactions (e.g., sorption) at the mineral‐organic matter interface that have different consequences for soil carbon stabilization and persistence. Strong interactions with mineral surfaces can increase soil carbon preservation and decrease CO_2_ emissions.^[^ ^6^, ^33^ ^]^ Nevertheless, there is limited evidence of the associations between MP‐DOM and soil minerals.

In this study, we aimed to (1) compare the bioavailability and characteristics between MP‐DOM and NOM and (2) estimate their different contribution to the soil mineral‐associated carbon pool. We hypothesized that the relatively bioavailable and labile MP‐DOM would stimulate microbial respiration. Additionally, we hypothesized that the selective preservation of minerals on MP‐DOM would weaken the formation of mineral‐associated organic carbon (MAOC).

To test our hypotheses, we used microcosm experiments with natural DOM reference materials and DOM derived from both conventional and biodegradable microplastics. Two petroleum‐based plastics, polyethylene (PE) and polyvinyl chloride (PVC), and two bio‐based plastics, polybutylene adipate terephthalate (PBAT) and polylactic acid (PLA), were studied due to their widespread use.^[^ ^34^, ^35^ ^]^ We compared these microplastics to two international reference materials for NOM: Suwannee River NOM (SRNOM) and Pahokee Peat Humic acid (PPHA). To eliminate the influence of indigenous soil organic matter, we used diluted soil suspension as a microbial inoculum, and quartz sand and two typical soil minerals (kaolinite and goethite) as microcosm matrices. Kaolinite and goethite are widely present in soils and play vital roles in protecting soil carbon.^[^ ^36^ ^]^ The CO_2_ emissions and formation of MAOC in each microcosm were determined. To estimate the mechanisms underlying differences in microbial respiration and MAOC formation, we compared the spectroscopic and molecular fingerprints of MP‐DOM versus NOM by using fluorescence excitation‐emission matrix spectroscopy (EEM) and ultrahigh‐resolution Fourier transform ion cyclotron resonance mass spectrometry (FT‐ICR‐MS), respectively. We estimated the sorption characteristics of organic matter onto the two minerals (kaolinite and goethite) via adsorption kinetic experiments to ensure the selective preservation of minerals. Our study advances the understanding of how and why microplastic pollution impacts soil carbon mineralization and sequestration.

## 2 Results

### 2.1 Production of CO_2_ and MAOC

**Figure 1:**
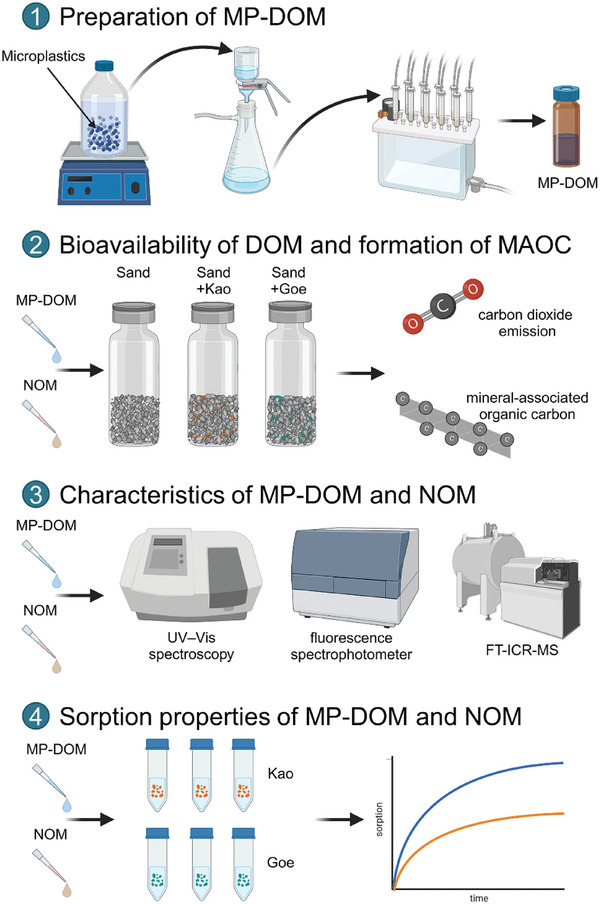
Overview of the experiment design. 1) preparation of microplastic‐derived DOM (MP‐DOM); 2) experimental flow for estimating bioavailability of DOM and formation of MAOC; 3) optical and molecular characteristics of MP‐DOM and natural DOM; 4) experimental design for determining sorption properties of MP‐DOM and natural DOM.

After 21 days of incubation, cumulative CO_2_ emissions were far higher from MP‐DOM than those from the NOM treatments (**Figure** **1**a). In the PLA‐DOM and PBAT‐DOM treatments, the mean ± standard deviation for the generation of CO_2_ was 160.96 ± 9.81 and 202.84 ± 14.23 mg C g^−1^ DOC, respectively, which was higher than in SRNOM and PPHA treatments of 29.14 ± 6.91 and 50.69 ± 9.26 mg C g^−1^ DOC, respectively (F_5,12_ = 50.59, *p* < 0.001). The cumulative CO_2_ emissions in PE‐DOM and PVC‐DOM were also greater than those in NOM treatments with 62.31 ± 5.21 and 61.08 ± 8.20 mg C g^−1^ DOC, respectively (F_5,12_ = 50.59, *p* < 0.001). The larger CO_2_ fluxes of the MP‐DOM may be attributed to it potentially having the most bioavailable molecules.

In parallel, larger MAOC pools were generated in microcosms inoculated with NOM (Figure 1b). The amounts of MAOC in SRNOM and PPHA ranged from 44.73 to 96.73 µg g^−1^ minerals, which were 1.50–5.84 fold higher than those in the MP‐DOM treatments (F_5,12_ = 14.20, *p* < 0.001 for kaolinite; F_5,12_ = 15.27, *p* < 0.001 for goethite). Together, these observations indicate that NOM may be more likely to sorb directly to the minerals and form strong organo‐mineral bonds than MP‐DOM. As expected, the addition of kaolinite and goethite decreased the decomposition and mineralization of MP‐DOM and NOM, and thus cumulative CO_2_ emissions by 17–42% (F_5,12_ = 14.57, *p* < 0.001) and 32–69% (F_5,12_ = 11.55, *p* < 0.001), respectively (Figure 1a).

### 2.2 Comparison between MP‐DOM and NOM Characteristics

**Figure 2:**
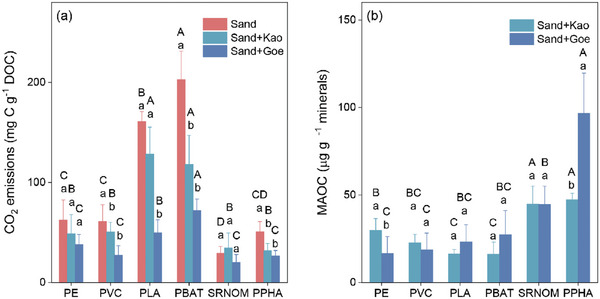
Microplastic‐derived DOM emits more carbon dioxide and stores less carbon than NOM. Cumulative CO_2_ emissions a) and MAOC formation b) of MP‐DOM and NOM after three weeks of incubation. Bars are means and lines are standard deviations. Different uppercase letters indicate statistically significant differences (*p* < 0.05) between DOM types in the same matrix of either sand, sand with kaolinite (Kao), or sand with goethite (Geo). Different lowercase letters indicate statistically significant differences (*p* < 0.05) between mineral treatments in each DOM microcosm. PE, polyethylene‐derived DOM; PVC, polyvinyl chloride‐derived DOM; PLA, polylactic acid‐derived DOM; and PBAT, polybutylene adipate terephthalate‐derived DOM.

To determine whether the higher CO_2_ emissions from MP‐DOM arose because of a greater bio‐lability, we compared the spectroscopic and molecular signatures of MP‐DOM with NOM. In comparison with the four MP‐DOM treatments, NOM showed a higher level of aromaticity, humification, and polarity with evidence of fewer microbially derived molecules. The spectroscopic indices of SUVA_254_ (F_5,12_ = 1697.14, *p* < 0.001), E253/E203 (F_5,12_ = 61.67, *p* < 0.001), FI (F_5,12_ = 523.03, *p* < 0.001), and HIX (F_5,12_ = 7077.28, *p* < 0.001) were all higher in the NOM compared to MP‐DOM treatments, whereas BIX values were lower (F_5,12_ = 642.73, *p* < 0.001) (**Figure** **2**). The SUVA_254_ and E253/E203 values were 2.85 ± 0.02 L mg C^−1^ m^−1^ and 0.53 ± 0.02 for SRNOM and 4.08 ± 0.15 L mg C^−1^ m^−1^ and 0.60 ± 0.01 for PPHA, respectively, while the values for the four MP‐DOM treatments only ranged from 0.03 to 0.30 L mg C^−1^ m^−1^ and 0.19 to 0.30 for SUVA_254_ and E253/E203, respectively. The parallel factor analysis (PARAFAC) model based on EEM data also identified different proportions of humic‐like and protein‐like components between MP‐DOM and NOM, further suggesting that MP‐DOM was more labile. In the MP‐DOM, PARAFAC identified a humic‐like component (C1), natural fulvic component (C2), and two protein‐like components (C3 and C4) (Figure S4 and Table S3, Supporting Information). C3 dominated in the conventional and biodegradable microplastic‐derived DOM, accounting for 42 ± 6% of PBAT‐DOM, 34 ± 5% of PLA‐DOM, and 100 ± 1% of both PE‐DOM and PVC‐DOM, respectively. For the two NOM treatments (Figure S5 and Table S4, Supporting Information), PARAFAC identified two humic‐like components (NC1 and NC2) and a quinone‐like component (NC3), with NC1 and NC2 together accounting for 90 ± 2% and 66 ± 3% of the relative abundances of components in SRNOM and PPHA, respectively.

**Figure 3:**
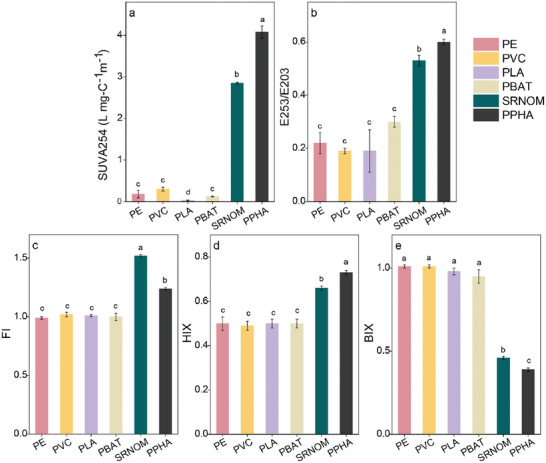
Optical properties differ between MP‐DOM and NOM. The values of a) SUVA254, b) E253/E203, c) fluorescence index, FI, d) humification index, HIX, and e) biological index, BIX. Bars are means and lines are standard deviations. Different lowercase letters indicate statistically significant differences (*p* < 0.05) between different DOM types.

To estimate further the differences between MP‐DOM and NOM, we identified molecular characteristics using FT‐ICR‐MS (Table S5, Supporting Information). MP‐DOM contained fewer DOM formulas (ranging from 920 to 3201) compared to NOM (4132 and 4695 for SRNOM and PPHA, respectively), and was less recalcitrant, as evidenced by lower values of AI_w_, DBE_w_, and NOSC_w_ and higher values of H/C_w_ (Table S5, Supporting Information). Additionally, there were more labile‐like compounds in the four MP‐DOM treatments, as shown by lower O/C and higher H/C values (**Figure** **3**). The mean ± standard deviation for the MLB_L_ values in the four MP‐DOM were 0.50 ± 0.02, 0.48 ± 0.04, 0.29 ± 0.08, and 0.60 ± 0.07 for PE‐, PVC‐, PLA‐, and PBAT‐DOM, respectively, compared with 0.04 ± 0.01 and 0.11 ± 0.02 observed for SRNON and PPHA, respectively (F_5,12_ = 67.604, *p* < 0.001). We further found that MP‐DOM was dominated by smaller molecular mass and more bioavailable compound classes. Lignin/phenolic‐like compounds were the most abundant in PE‐ and PVC‐DOM, accounting for 34.2 ± 2.3% and 42.5 ± 6.2% of molecular formulae, respectively, followed by N‐less aliphatic‐like and carbohydrate‐like compounds. For PBAT‐ and PLA‐DOM, the most abundant subcategory was N‐less aliphatic‐like compounds (86.1 ± 6.4% and 71.8 ± 6.7% of formulae, respectively). The percentages of polycyclic aromatic‐like and aromatic‐like compounds were minor in MP‐DOM, i.e., <6.8%. In contrast, polycyclic aromatic‐like and aromatic‐like compounds together accounted for 16.3 ± 2.3% and 48.6 ± 4.7% of formulae in SRNOM and PPHA, respectively, which were higher than those in MP‐DOM (2.1% to 6.8%) (Figure 3).

### 2.3 Comparison of Sorption Behaviors between MP‐DOM and NOM

**Figure 4:**
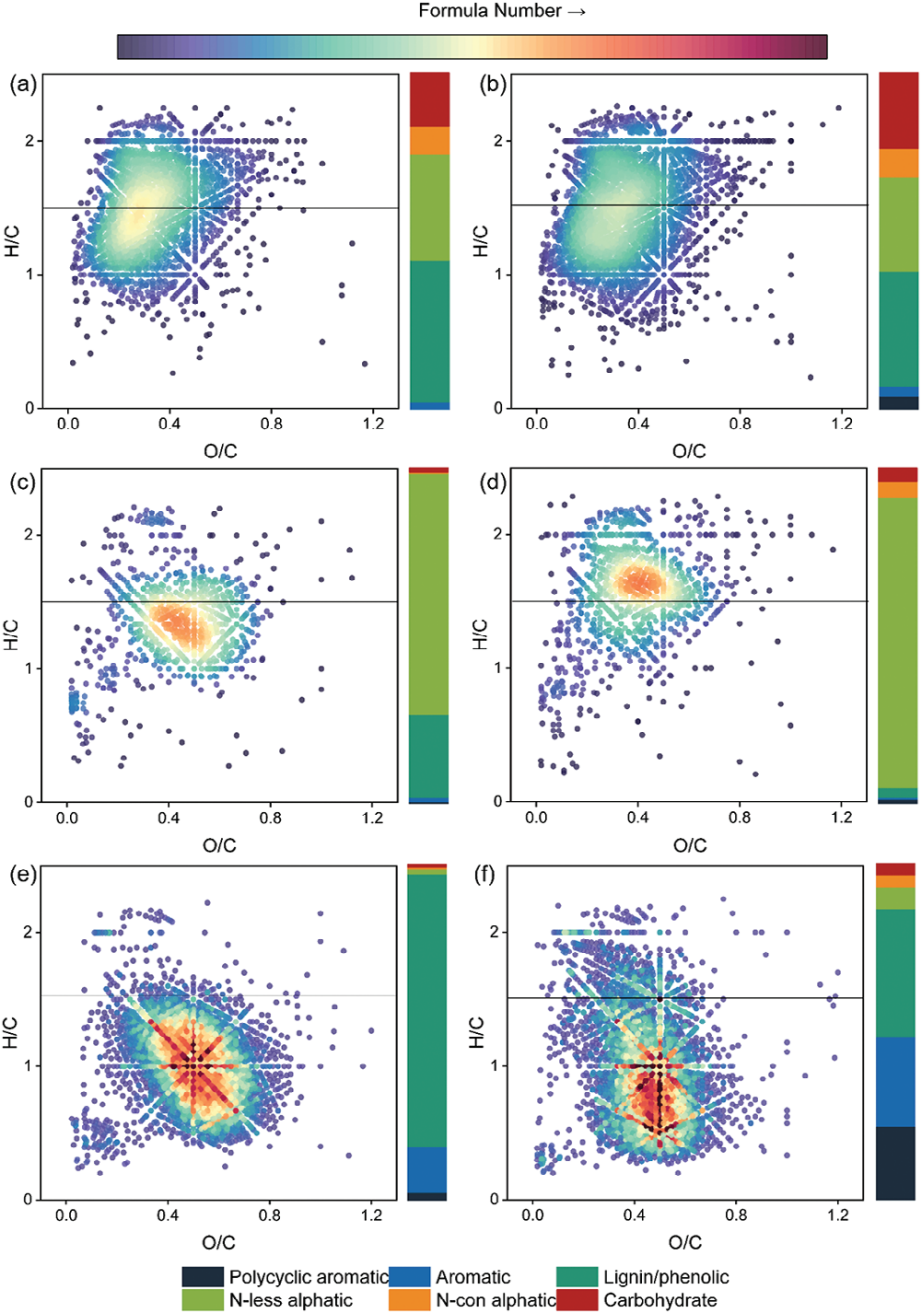
Microplastics leach more molecular formulae with a high index of lability. Van Krevelen diagram of MP‐DOM and NOM, PE‐DOM a), PVC‐DOM b), PLA‐DOM c), PBAT‐DOM d), SRNOM e), and PPHA f). Points are individual molecular formulae, with those above the horizontal line indicating greater lability (i.e., H/C > 1.5). The stacked bar plot on the right side of each Van Krevenlen panel represents molecular composition based on relative intensity in six compound classes. The heatmap bar represents the formula number, and a darker red color indicates a greater density of molecular formulae. [Correction added on 9 January 2025, after first online publication: figure 4 and 5 were interchanged, now corrected.]

We found a higher affinity for mineral protection of NOM than MP‐DOM, partly explaining why MP‐DOM was more readily mineralized as CO_2_. The adsorption of all the DOM on kaolinite and goethite increased rapidly and reached equilibrium within 8 h, but the equilibrium concentrations of SRNOM and PPHA on the minerals were higher (F_5,12_ = 189.20, *p* < 0.001 for kaolinite; F_5,12_ = 1664, *p* < 0.001 for goethite) than for MP‐DOM (**Figure** **4**). At equilibrium, the mean ± standard deviation for the concentrations of SRNOM and PPHA were 2.22 ± 0.02 and 1.41 ± 0.13 mg g^−1^ on kaolinite and 3.67 ± 0.12 and 4.06 ± 0.05 mg g^−1^ on goethite, respectively, whereas the concentrations of MP‐DOM only ranged from 0.25 to 0.98 mg g^−1^ on the minerals. This observation may have arisen because compounds in NOM had greater affinities with minerals than MP‐DOM compounds. Formulae with high molecular weight and polarity, and rich in oxygen and aromatic moieties, as we observed for NOM (Figures S10–S13, Supporting Information), generally have stronger affinity to minerals and are preferentially adsorbed.^[^ ^37^, ^38^ ^]^ In parallel, we also observed different DOM adsorption behaviors between goethite and kaolinite, with 23.2–65.2% more DOM sorbed on goethite (Figure 4). The increased sorption on goethite was particularly evident for NOM (39.6–65.2%) in comparison with MP‐DOM (23.2–32.4%). Previous studies indicated carboxylic functional groups play a significant role in the sorption of DOM on goethite,^[^ ^38^ ^]^ and we confirmed that polar functional groups were more abundant in NOM than in MP‐DOM.

To identify the mechanism explaining why MP‐DOM adsorbed less to minerals, we measured the Zeta potentials of the two minerals and six DOM treatments (Figure S6, Supporting Information). Although all the DOM samples and kaolinite had negative charges at pH 7.0, the goethite was positively charged. Thus, the goethite surface was favorable for the adsorption of negatively charged DOM via electrostatic attraction.^[^ ^39^ ^]^ Fewer negative charges were observed for MP‐DOM than NOM (F_5,12_ = 27.02, *p* < 0.001), which may be also the reason that MP‐DOM was adsorbed less by goethite. We also observed a higher proportion of micropores (<2 nm) on goethite than kaolinite (Figure S1, Supporting Information). Previous studies indicated that micropores were preferentially covered with DOM compared to other parts of pores (mesopores and macropores),^[^ ^40^ ^]^ which may be another reason for the greater DOM sorption on goethite. Additionally, both goethite and kaolinite had hydroxyl sites (Figure S7, Supporting Information), which could sorb DOM via ligand exchange.^[^ ^41^ ^]^

Changes in DOM characteristics after sorption further explained the role of minerals in mediating the bioavailability of MP‐DOM. For MP‐DOM, no fluorescent signal was detected after the DOM was sorbed by kaolinite and there were small (<5%) changes in the relative abundances of PARAFAC components after sorption on goethite (Figures S8,S9, Supporting Information). Likewise, the more bioavailable PARAFAC components in NOM generally decreased after sorption on the two minerals, especially on goethite. For instance, the relative abundances of quinone‐like NC3 was reduced from 10.2% to 6.9% in SRNOM and from 34.1% to 16.1% in PPHA after sorption on goethite (Figure S5, Supporting Information). Using FT‐ICR‐MS, we found that the relative abundances of molecules with lower *m*/*z*, O/C, and DBE values, and higher H/C values all generally increased after sorption (Figures S10–S13, Supporting Information). Additionally, both minerals showed high affinity for more oxidized and less bioavailable molecules with larger mass, which were more characteristic of NOM than MP‐DOM. The less sorption of more bioavailable components in MP‐DOM may contribute to the higher CO_2_ emissions.

**Figure 5:**
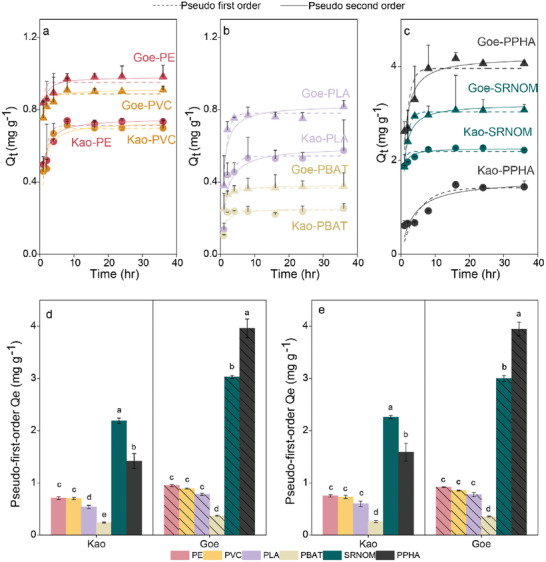
Higher sorption of MP‐DOM than NOM on minerals. Adsorption kinetics of different DOM on kaolinite (Kao) and goethite (Goe); a) conventional MP‐DOM; b) biodegradable MP‐DOM; and c) NOM. Qt means the sorbed amount of DOM at time *t*. Pseudo‐first‐order (dot line) and Pseudo‐second‐order (solid line) models were used to fit the sorption data. d) The maximum adsorption capacity based on the Pseudo‐first‐order model, Bars are the modeled equilibrium concentration on minerals, and lines are the modeled standard deviations. e) The maximum adsorption capacity based on the Pseudo‐second‐order model, Bars are the modeled equilibrium concentration on minerals, and lines are the modeled standard deviations.

**Figure 6:**
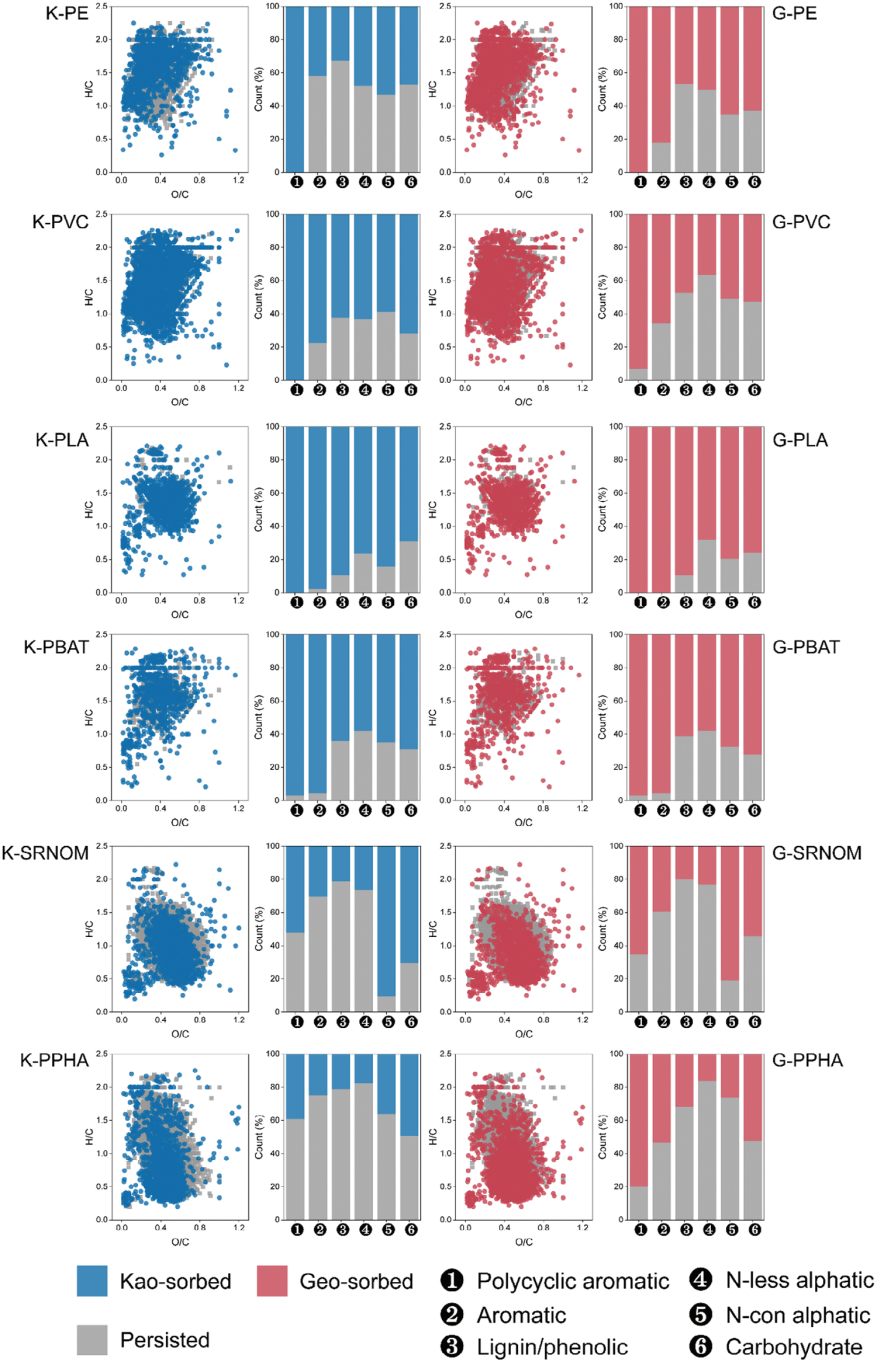
Van Krevelen diagrams and bar graphs exhibit the adsorbed and not adsorbed DOM molecules by kaolinite and goethite. Blue dots are kaolinite‐sorbed molecules, red dots are goethite‐sorbed molecules, and the grey dots are the molecules dissolved in solutions. K‐, Kaolinite, G‐, Goethite, PE, PE‐DOM, PVC, PVC‐DOM, PLA, PLA‐DOM, and PBAT, PBAT‐DOM.

**Figure 7:**
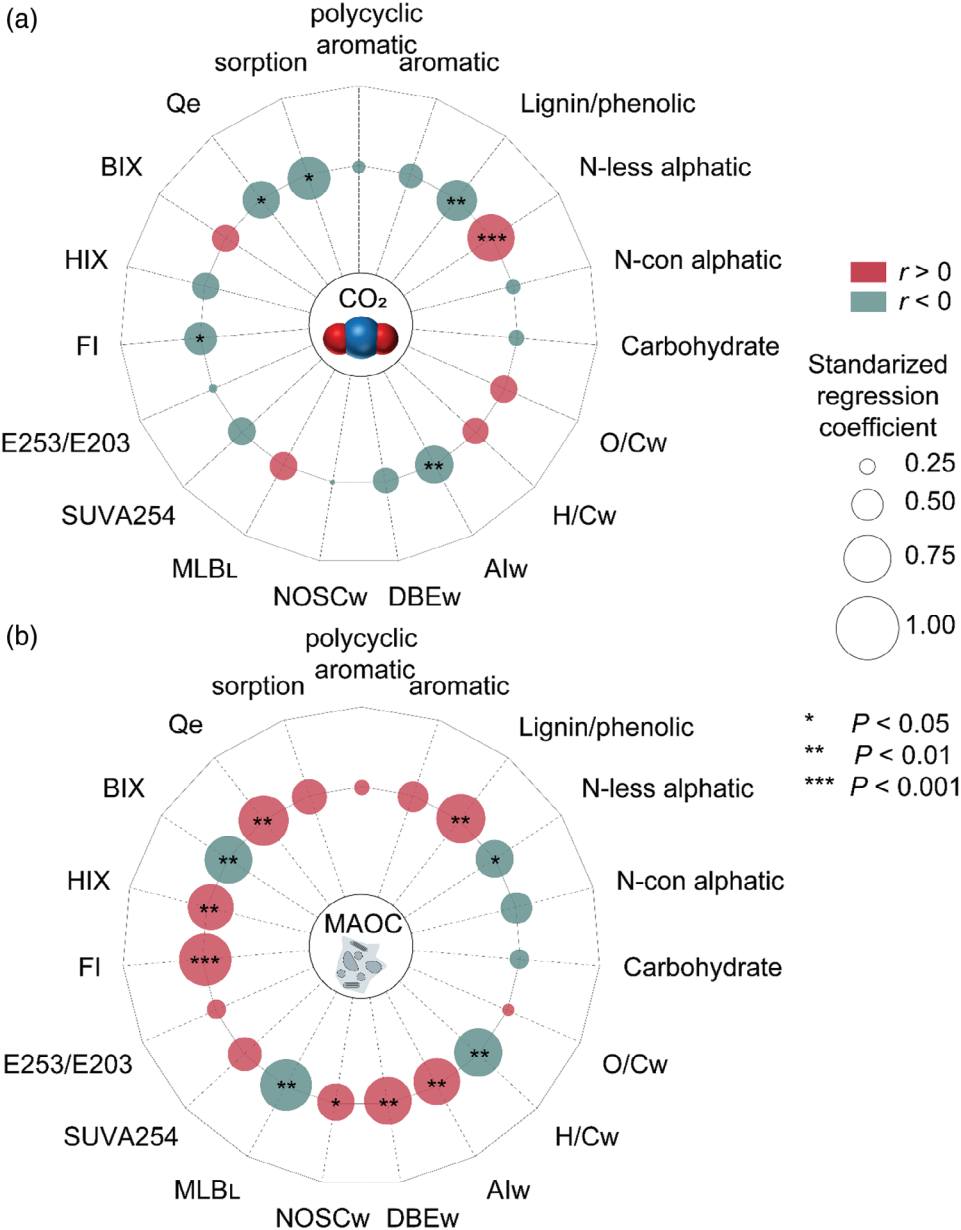
Relationship between the DOM parameters and the generations of CO_2_ and MAOC. Mantel test based on Euclidean distance was carried out. Edge width corresponds to the Mantel's r value, and the edge color denotes the statistical significance. Pairwise correlations of the parameters are shown with a color gradient denoting Pearson's correlation coefficients. DOM parameters include the relative abundances of the six subcategories (polycyclic aromatic‐like, aromatic‐like, lignin/phenolic‐like, N‐less aliphatic‐like, N‐con aliphatic‐like, and carbohydrate‐like), formula number, molecular weight (M/Z, intensity‐weighted), oxygen carbon ratio (O/C, intensity‐weighted), hydrogen carbon ratio (H/C, intensity‐weighted), modified aromaticity index (AI, intensity‐weighted), double bond equivalence (DBE, intensity‐weighted), nominal oxidation state of carbon (NOSC, intensity‐weighted), percentage of labile‐like compounds (MLB_L_), specific ultraviolet absorbance at 254 nm (SUVA_254_), ratio of absorbance at 253 to 203 nm (E253/E203), fluorescent index (FI), humic index (HIX), biological index (BIX), the equilibrium concentration of DOM on minerals (Qe), and the sorbed percentage of DOM on minerals (sorption).

We further analyzed molecules that were sorbed onto minerals versus remained in DOM after the sorption, and discovered more bioavailable molecules that could increase microbial CO_2_ emissions in NOM treatments were preferentially protected by minerals. For both conventional and biodegradable microplastics, most polycyclic aromatic‐like and aromatic‐like compounds were sorbed onto kaolinite and goethite, whereas only parts of these formulae were sorbed for NOM (**Figure** **5**). In the NOM treatments, much more bioavailable compounds, such as N‐containing aliphatic molecules in the SRNOM treatment and carbohydrate‐like molecules in the PPHA treatment, were preferentially sorbed (Figure 5). Overall, the mean ± standard deviation for number of recalcitrant formulae (with a H/C ratio < 1.5) sorbed onto minerals in the NOM treatments (825 ± 251) was larger (F_5,12_ = 125.4, *p* < 0.05) than those in the MP‐DOM (343 ± 125). After mineral sorption, the overall number of recalcitrant formulae left in the solution was 2512 ± 124 in the NOM treatments versus 232 ± 152 in the MP‐DOM (F_5,12_ = 421.5, *p* < 0.05), whereas the number of bioavailable formulae (with a H/C ratio ≥ 1.5) left after mineral sorption were 292 ± 27 in the NOM treatments versus 418 ±76 in the MP‐DOM treatments (F_5,12_ = 98.4, *p* < 0.05). The higher number of bioavailable formulae in the MP‐DOM treatments occurred despite there being far fewer formulae in total (1885 ± 648) than in the NOM (4413 ± 203). These results suggest that the selective sorption of molecules onto minerals contributes to differences in the generation of CO_2_ and MAOC among different types of DOM (**Figures** **6** and **7**).

## 3 Discussion

**Figure 8:**
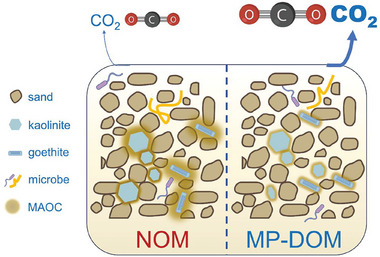
Conceptual paradigm depicting the distinct carbon processes between MP‐DOM and NOM. MP‐DOM induced greater CO_2_ emissions and lower carbon storage than NOM because of its higher lability and lower sorption to minerals.

Our results collectively indicated that MP‐DOM could be more easily biodegraded and more labile than NOM because it had different molecular characteristics. Aromatic‐like and lignin/phenolic‐like molecules with high oxidation state, double bonds/rings, and aromatization were the most abundant formulae in SRNOM and PPHA, whereas microplastic‐derived DOM contained more N‐less aliphatic‐like and carbohydrate‐like compounds with low oxidation state, double bonds/rings and aromatization. It has traditionally been assumed that small (low molecular weight) and aliphatic‐like DOC compounds are more preferentially degraded by microbes than aromatic‐like compounds.^[^ ^42^, ^43^ ^]^ Preferential consumption of more labile compounds, especially by copiotrophs (*r*‐strategists) characterized by rapid growth and reproductive rates, are in turn positively related to CO_2_ emissions.^[^ ^44^, ^45^ ^]^ For these reasons, MP‐DOM contributed to the higher levels of CO_2_ emissions (**Figure** **8**). Additionally, the contrasting molecular composition suggests that MP‐DOM and NOM should exhibit distinct environmental fates and behaviors, further altering their interactions with soil minerals.

We found clear differences in carbon sorption between NOM and MP‐DOM, wherein the latter induced lower carbon storage than NOM because of its lower sorption to minerals. Compared to MP‐DOM, molecules in the more humified and recalcitrant NOM interact more strongly with minerals and so should persist for longer periods in the environment. These conclusions were supported by correlating both CO_2_ emissions and rates of MAOC formation with characteristics of DOM. Aliphatic‐like DOM molecules are known to be preferentially degraded by microbes,^[^ ^42^ ^]^ which can result in higher CO_2_ emissions. The molecular indices, including O/C and H/C ratios, fluorescent parameters, and equilibrium sorption mass were also closely correlated with MAOC formation (Figure 7; Figure S15, Supporting Information). Molecules with higher O/C ratios and lower H/C ratios were preferentially sorbed by minerals (Figures S12,S13, Supporting Information) and so correlated positively with MAOC formation. Similar mechanisms may also explain the differences in carbon mineralization between biodegradable and conventional microplastic treatments. In the current study, DOM derived from biodegradable microplastics induced greater CO_2_ emissions than that from conventional microplastics. The labile compounds, including carbohydrate‐like, N‐less aliphatic, and N‐containing aliphatic compounds, were more abundant in biodegradable microplastic‐derived DOM (Figure 4), which would be more preferentially degraded by microbes. Lower sorption of biodegradable microplastic‐derived DOM on minerals was observed (Figure 5). For these reasons, the treatments with biodegradable microplastic‐derived DOM emitted higher levels of CO_2_ in comparison with conventional microplastic‐derived DOM.

Although we did not consider the responses of microorganisms explicitly in this study, previous studies have indicated that MP‐DOM may foster greater microbial activity and growth in aquatic environments than NOM,^[^ ^26^, ^28^ ^]^ and similar processes should be expected in soils.^[^ ^46^, ^47^ ^]^ Therefore, the greater CO_2_ emissions in MP‐DOM treatments may be also due to the induced microbial activity or increased microbial biomass. The alternations in microbial community composition (e.g., oligotrophy vs copiotrophy) may also contribute to the changes in CO_2_ emissions. Further understanding of how microbial processes respond and adapt to MP‐DOM, including microbial abundance, phenotypic activity, carbon utilization, and community composition, will be necessary to predict the consequences of plastic pollution for carbon cycling. Additionally, prior research suggests that microplastics in soil can create a more aerobic environment, increasing oxygen availability and potentially influencing labile carbon dynamics.^[^ ^48^, ^49^ ^]^ The impact of this induced aerobic microenvironment on soil DOM quantity and quality warrants further investigation.

Terrestrial microplastic pollution, if unmitigated, is projected to double over the next three decades,^[^ ^14^ ^]^ and our results suggest that this increase may have broad implications for soil carbon fluxes and climate change. ≈12 000 million tons of plastics are estimated to enter the natural environment by 2050.^[^ ^15^ ^]^ If all these plastics are converted into soil microplastics, we estimate that between 1.5–81 million tons of CO_2_ would be emitted from MP‐DOM in <2 months, given our range of DOC leaching (0.55–9.20 mg C g^−1^ plastics) and mineralization rates (6–20%). This estimate is comparable to some of the largest anthropogenic CO_2_ emissions, such as from the steel and (1000 million tons in 2050) cement (280 million tons in 2050) industry.^[^ ^50^ ^]^ Additionally, plastic end‐of‐life emissions would lead to ≈375 million tons of CO_2_ equivalent (CO_2_e) greenhouse gas emissions in 2050 (Table S7, Supporting Information). It is currently challenging to accurately quantify the contribution of MP‐DOM to atmosphere CO_2_ with the data available at present. Nevertheless, this study emphasizes the importance of improving assessments of the carbon footprint of plastics, including MP‐DOM considerations. Our study highlights that the multiple aspects associated with the influence of management on plastic waste need to be consider in an integrative manner, further mitigating its impacts on soil carbon pool.

Finally, biodegradable plastics have long been viewed as sustainable alternatives to conventional petroleum‐based polymers. However, as revealed in this study, the biodegradable microplastics released nearly 17‐times more DOC and induced 3‐times more CO_2_ emissions than the conventional microplastics, partly because they had less chemical bonding and physical protection by soil minerals. Given that in‐soil degradation would be one of the main intended end‐of‐life scenarios of biodegradable plastics, greenhouse gas emissions may therefore already be elevated in regions where biodegradable polymers have been heavily used and abandoned. Furthermore, using the understanding generated here of how minerals affect CO_2_ emissions and MAOC formation can help mitigate carbon emissions from soils. Previous studies suggest that mineral protection is a more significant factor in regulating subsoil carbon decomposition, while microbial activity dominates topsoil carbon turnover.^[^ ^51^ ^]^ Additionally, minerals have been widely used as soil conditioners in agriculture due to their excellent sorption and buffering capacity;^[^ ^52^, ^53^ ^]^ therefore, our results potentially suggest that the minerals offer promise to counteract the negative consequences of subsoil microplastic pollution for carbon storage and climate change. What we should notice is that this study is based on limited microbial and mineral conditions, which may lead to potentially biased observation. Further studies with different soils are necessary to ensure the broader representativeness of our findings.

## 4 Conclusion

Microplastics not only pose a substantial threat to the environment but also have the potential to exacerbate climate change by contributing to greenhouse gas emissions. Our findings provide compelling experimental evidence that MP‐DOM, due to its higher lability and lower mineral sorption, significantly impacts carbon cycling by promoting CO_2_ emissions and hindering carbon sequestration compared to NOM. Specially, biodegradable microplastics‐derived DOM have a more pronounced positive effect on soil CO_2_ emissions. This study makes a significant contribution to our understanding of the intricate relationship between microplastics and climate change. By elucidating the role of plastics in climate processes, this research provides critical scientific evidence to inform effective policy development and environmental management strategies.

## 5 Experimental Section

### Microplastics, Minerals, and other Materials

Conventional (PE and PVC) and biodegradable (PLA and PBAT) microplastics were purchased from the Aladdin (Shanghai, China) and XingWang Plastic (Dongguan, Guangzhou, China), respectively. Microplastic particles (white powders) were sieved to the size range of 150–180 µm (80−100 mesh), cleaned with ultrapure water, air‐dried in the fume hood, and stored at 4 °C before use. The functional groups of the microplastics were determined by Fourier transform infrared spectroscopy (FTIR, IS5, Semefei, USA; see Figure S1, Supporting Information). SRNOM (catalog number 2R101N) and PPHA (catalog number 1S103H) were purchased from the International Humic Substances Society (https://humicsubstances.org/).

Kaolinite and goethite powders were purchased from Aldrich (Shanghai, China) and Sigma‐Aldrich (St. Louis, Missouri, USA), respectively. X‐ray diffraction (XRD, D8 Advance, Bruker, Germany) and FTIR spectroscopy were used to confirm the characteristic peaks of these two minerals.^[^ ^54^, ^55^ ^]^ The isoelectric point of goethite was previously reported as being close to ∼9.0–9.4, whereas kaolinite has an isoelectric point of ∼2.7–4.1 for the overall surface and ∼7.3 for the surface edges.^[^ ^56^ ^]^ Nitrogen Brunauer‐Emmett‐Teller (BET) and Barrett‐Joyner‐ Halenda (BJH) analysis revealed that the mean ± standard deviation specific surface areas (SSA) and pore volumes (PV) were 15.99 ± 0.04 m^2^ g^−1^ and 0.12 cm^3^ g^−1^ for kaolinite, and 12.27 ± 0.11 m^2^ g^−1^ and 0.08 cm^3^ g^−1^ for goethite, respectively (Figure S1 and Table S1, Supporting Information).

Experimental soil was collected from the Shenmu Erosion and Environment Experimental Station in Shaanxi province (38.7853 °N, 110.3616 °E). The soil had a mean pH ± standard deviation of 7.99 ± 0.03, and contained 2.84% clay, 64.29% silt, and 32.87% sand. The soil carbon and nitrogen contents (mean ± standard deviation) were 1.90 ± 0.01 and 0.01 ± 0.00 mg kg^−1^ dry soil, respectively. The soil was used due to the extensive areas in China. For preparing inoculum, the soil was sieved through 4 mm, and 100 g equivalent dry mass soil was mixed with 100 mL sterile distilled water with a waring blender under sterile conditions. After centrifugation at 1000 g for 10 min, the soil suspension was diluted 100 times and used for incubating the microcosms.^[^ ^57^ ^]^

### Preparation of MP‐DOM

≈18.0 g of microplastic particles and 900 mL of sterilized ultrapure water were added into a 1 L sterilized amber glass bottle for 30 days under dark conditions.^[^ ^28^ ^]^ All the bottles were mixed under magnetic stirring at room temperature of ≈25 °C. On days 3, 7, 14, 21, and 30, ≈20 mL of each mixture was filtered through a 0.45 µm cellulose acetate membrane to obtain the microplastic leachates, and the dissolved organic carbon (DOC) concentration, UV‐vis absorption spectra, and EEM fluorescence spectra of the solution were all measured as described below. After leaching for 30 days, the remaining mixture was again filtered through a 0.45 µm cellulose acetate membrane filter to obtain the microplastic‐derived DOM (that is, PE‐DOM, PVC‐DOM, PLA‐DOM, and PBAT‐DOM) used for incubations comparing MP‐DOM and NOM. The MP‐DOM was further processed using a solid phase method, and the molecular characteristics were estimated using FT‐ICR‐MS described below.

### Bioavailability of DOM and Formation of MAOC

To compare bioavailability and MAOC formation between MP‐DOM and NOM, a laboratory incubation experiment were conducted (Figure S2, Supporting Information). Microcosms consisted of three different matrices: (i) sterilized quartz sand (600–800 µm) with 5% kaolinite (w/w), (ii) sterilized quartz sand with 5% goethite (w/w), and (iii) only sterilized quartz sand. The addition rate of minerals was based on the properties of clay minerals in loess soil in China.^[^ ^36^ ^]^ All the matrices were homogenized on a rolling mixer for 1 h. Four MP‐DOM and two NOM were thoroughly dissolved in deionized water and prepared at ≈250 mg C L^−1^. A 2 mL aliquot of each DOM source was homogeneously added into 10 g of the matrix (0.05 mg C g^−1^ soil), and the mixture was pre‐incubated for 36 h at 25 °C to allow equilibration between DOM and the minerals. After the pre‐incubation, the soil inoculum suspension (100 µL) was added. This inoculum suspension contained 2.98 mg C L^−1^, thus, a negligible amount of carbon was added via the inoculum. Additionally, to promote microbial anabolism and eliminate nutrient limitations, 100 µL of NH_4_Cl and NaH_2_PO_4_ solution were added to the incubated matrix in a final C:N:P ratio of 100:10:1.

A total of 63 microcosms were constructed with 3 minerals (no mineral, kaolinite, and goethite) × 7 DOM treatments (4 MP‐DOM, two NOM treatments, and a water only control), each replicated three times. The microcosms were maintained gastight at 25 °C and 60% humidity in the dark (Figure S2, Supporting Information). On days 7, 14, and 21, a gas sample was collected from the headspace of the bottle using a gastight needle. The CO_2_ concentrations in the headspace samples were measured using a gas chromatograph (Clarus 680, PerkinElmer, US) with a thermal conductivity detector to quantify the biodegradability of DOM. The injector and detector temperatures were 200 and 250 °C, respectively. The oven temperature program was set as: 40 °C for 10 min, 40 to 160 °C at 40 °C/min^−1^, and 160 °C for 2 min. The CO_2_ concentrations in the water blank treatment were used to eliminate background interference. Emissions were calculated as the difference in concentration between the DOM treatments and the blank treatment at each sampling point, and cumulative CO_2_ emissions, i.e., the sum of emissions at each sampling point, were obtained. After the incubation, the matrix was collected and freeze‐dried. The MAOC in each treatment was quantified as the carbon content in the mineral matrix after wet sieving (<53 µm)^[^ ^58^ ^]^ (Text S1, Supporting Information).

### Characteristics of MP‐DOM and NOM

The organic carbon concentration in DOM was determined with a TOC analyzer (Vario TOC, Elementar, Germany). A zeta potential meter (Zetasizer Nano ZS90, Marvin Limited, UK) was used to characterize the zeta potential of DOM at the same pH (≈7.0). UV‐vis spectroscopy (TU‐1900, General analysis, China) absorption (from 200 to 400 nm) was measured in a 10‐mm quartz cuvette with ultrapure water as blank. Specific ultraviolet absorbance was then calculated at 254 nm (SUVA_254_) as the ratio of absorbance at 254 nm to the DOC concentration, with a higher SUVA_254_ usually indicating a higher DOM aromaticity.^[^ ^59^ ^]^ The E253/E203 ratio was also calculated, an indicator of the types and numbers of substituents (e.g., ─COOH, ─OH, and ─C═O) on the benzene ring.^[^ ^60^ ^]^ A high E253/E203 value indicates that polar functional groups are the main substituent groups in DOM.

The EEM fluorescence spectra were measured using a fluorescence spectrophotometer (F97Pro, Lengguang, China) with a 1 cm quartz four‐pass‐dish. The excitation (Ex) and emission (Em) wavelength ranged from 200 to 500 nm (5 nm increment) and 250 to 600 nm (1 nm increment), respectively. Ex and Em slit widths were 10 nm and the scanning speed was 3000 nm min^−1^. Ultrapure water was used as a blank to eliminate the effects of Raman scatter.^[^ ^61^ ^]^ The fluorescence intensities were normalized to Raman units using the fluorescence intensity of the integrated ultrapure water Raman peak.^[^ ^62^ ^]^ Three fluorescent indices, including the fluorescent index (FI),^[^ ^63^ ^]^ humic index (HIX),^[^ ^64^ ^]^ and biological index (BIX),^[^ ^65^ ^]^ were calculated based on the EEM spectra, following the methods described in the SI Text S2 (Supporting Information). Parallel factor analysis (PARAFAC) was used to identify underlying chemical components in the DOM and applied to the EEM fluorescence spectral data using Matlab 2016a with the DOMFluor v.1.7 toolbox.^[^ ^66^ ^]^

The molecular composition of DOM was analyzed using FT‐ICR‐MS. Briefly, the DOM sample (acidified to pH 2.0 with HCl) was passed through a styrene‐divinylbenzene polymer cartridge (6 mL, 1 g, Bond‐Elut PPL, Agilent) by gravity at a rate of ≈ 1 mL min^−1^.^[^ ^67^ ^]^ The cartridge was then rinsed with 18 mL of acidified ultrapure water (pH 2.0) and dried under nitrogen. Methanol (12 mL) was used to elute the DOM from the cartridge. The elution was dried under nitrogen gas and reconstituted to 1.0 mL with methanol in a GC vial.^[^ ^68^ ^]^ Samples were measured with a Bruker SolariX FT‐ICR‐MS with a 15.0 T superconducting magnet with an ESI ion source in negative ion mode. Detailed instrument parameters are given in Text S2 (Supporting Information). The raw spectra were converted to a list of *m/z* values using Data Analysis software 4.2 (Bruker Daltonik GmbH, Bremen, Germany) with a signal to noise of >4 and a default intensity threshold of 100. Putative chemical formulas were assigned using software from the Environmental Molecular Sciences Laboratory based on the compound identification algorithm,^[^ ^69^ ^]^ described by Kujawinki and Behn^[^ ^70^ ^]^ and modified by Minor et al.^[^ ^71^ ^]^ The mass error for a given chemical formula was set <0.35 ppm. The DOM formulas were assigned to six groups based on the modified aromaticity index AI_mod_ and oxygen‐to‐carbon (O/C) and hydrogen‐to‐carbon (H/C) ratios:^[^ ^37^ ^]^ (1) polycyclic aromatic formulas (AI_mod_ > 0.66); (2) aromatic formulas (0.66 ≥ AI_mod_ > 0.50); (3) lignin‐like/phenolic formulas (AI_mod_ ≤ 0.50 and H/C < 1.5), (4) nitrogen‐less (N‐) aliphatic compounds (2.0 > H/C ≥ 1.5 and N = 0); (5) nitrogen‐containing (N+) aliphatic compounds (2.0 > H/C ≥ 1.5 and N > 0) and (6) carbohydrate‐like compounds (H/C ≥ 2.0 or O/C ≥ 0.9). The double bond equivalence (DBE), the proportion of labile‐like compounds (MLB_L_), and nominal oxidation state of carbon (NOSC) of each formula were also calculated.^[^ ^72^, ^73^, ^74^ ^]^ Weighted means of formula‐based molecular traits were calculated as the sum of the product of the trait value for each individual molecule and relative intensity divided by the sum of all intensities.^[^ ^75^ ^]^ All detailed calculations are in Text S2 (Supporting Information).

### Sorption Properties of MP‐DOM and NOM

Adsorption experiments between DOM and minerals were conducted following Lee and Hur^[^ ^56^ ^]^ (Table S2, Supporting Information). The initial pH of DOM solution was adjusted to 6.9–7.1 by 0.1 m HCl or 0.1 m NaOH, and no systematic shift was observed after the sorption experiment (Table S2, Supporting Information). ≈20 mL DOM solution (10 mg C L^−1^) and 15 mg kaolinite or goethite were added into a 50 mL glass vial. The vial was tightly capped and mixed horizontally at 120 rpm at 25 °C. Control samples with only DOM or only minerals were also included. Leachate from the minerals or loss of DOM was not observed (Table S2, Supporting Information). Triplicate samples were removed after 1, 2, 4, 8, 16, 24, or 36 h, and centrifuged at 2500 *g* for 15 min. The supernatant was filtered through a 0.45 µm syringe filter (polytetrafluoroethylene, PTFE), and the DOC concentration was measured. Two kinetic models, pseudo‐first‐order and pseudo‐second‐order models, were fitted to the data to compare sorption properties between MP‐DOM and NOM^[^ ^76^ ^]^ (Text S3, Supporting Information). To detect changes in DOM properties, UV‐Vis absorption spectra, EEM fluorescence spectra, and FT‐ICR‐MS were used to characterize the DOM after sorption. Additionally, to estimate changes in mineral surfaces, kaolinite and goethite before and after sorption were explored using FTIR (IS5, Semefei, USA) with the scanned wavenumber from 400 to 4000 cm^−1^.

### Statistical Analysis

The statistical significance between different treatments was evaluated by one‐way analysis of variance (ANOVA) followed by a post hoc Tukey's test. Pearson correlation coefficients among DOM parameters were estimated with the “psych” package in R.^[^ ^77^ ^]^ To explore the driving factors of CO_2_ emissions or MAOC formation, regression analyses were conducted to clarify the association between DOM parameters and CO_2_ emissions and MAOC formation after z‐score transformations of each variable. Significance for all analyses was determined at a probability level of *p* < 0.05. All the statistical analyses were performed in R (version 4.3.2, R Core Team, 2023).

## Conflict of Interest

The authors declare no conflict of interest.

## Author Contributions

J.W., X.W., and A.T. conceptualized the study idea; J.S. and Y.S. worked on the study design. All authors reviewed the manuscript and provided feedback on the study design, data analysis, and interpretation of results. All authors were responsible for the decision to submit the manuscript for publication.

## Supporting information



Supporting Information

## Data Availability

The data that support the findings of this study are available from the corresponding author upon reasonable request.
