# Supplementary material for: Microplastics Generate Less Mineral Protection of Soil Carbon and More CO2 Emissions
Source: Adv Sci (Weinh). 2024 Dec 30;12(7):2409585. doi: 10.1002/advs.202409585 (PMC11831443; doi:10.1002/advs.202409585)
Supplement: Supplementary file 1 — Supporting Information [file ADVS-12-2409585-s001.docx]

**Supporting Information for:**

**Microplastics result in less mineral protection of soil carbon and higher CO_2_ emissions**

Jia Shi ^a^, Andrew J. Tanentzap ^b^, Yuanze Sun ^c^, Jianjun Wang ^d^, Baoshan Xing ^e^, Matthias C. Rillig ^f^, Changchao Li ^g^, Ling Jin^g, h^, Fang Wang^i^, Tanveer M. Adyel^j^, Jianying Shang ^a^, Xiang Wang ^a, *^, Jie Wang ^c, *^

^a^ Key Laboratory of Arable Land Conservation (North China), College of Land Science and Technology, China Agricultural University, Beijing 100193, China

^b^ Ecosystems and Global Change Group, School of the Environment, Trent University, Peterborough K9L 0G2, Canada

^c^ Beijing Key Laboratory of Farmland Soil Pollution Prevention and Remediation, College of Resources and Environmental Sciences, China Agricultural University, Beijing 100193, China

^d^ State Key Laboratory of Lake Science and Environment, Nanjing Institute of Geography and Limnology, Chinese Academic of Sciences, Nanjing 210008, China

^e^ Stockbridge School of Agriculture, University of Massachusetts, Amherst, MA 01003, USA

^f^ Freie Universität Berlin, Institut für Biologie, Altensteinstrasse 6, 14195 Berlin, Germany

^g^ Department of Civil and Environmental Engineering, The Hong Kong Polytechnic University, Hung Hom, Kowloon, Hong Kong 999077, China

^h^ Department of Health Technology and Informatics, The Hong Kong Polytechnic University, Hung Hom, Kowloon, Hong Kong 999077, China

^i^ State Key Laboratory of Soil and Sustainable Agriculture, Institute of Soil Science, Chinese Academy of Sciences, Nanjing 210008, China

^j^ Bioscience and Food Technology Discipline, RMIT University, Melbourne, VIC 3000, Australia

* Corresponding author:

Xiang Wang

Email: [wangxiang@cau.edu.cn](mailto:wangxiang@cau.edu.cn)

Jie Wang

Email: [jiewangcau@cau.edu.cn](mailto:jiewangcau@cau.edu.cn)

**Summary**

Number of pages: 35

Number of figures: 15

Number of tables: 7

**Content**

**Test S1 Determination of MAOC**

**Text S2. DOM parameters calculation based on EEM and FT-ICR-MS.**

**Text S3. Empirical kinetic models**

**Table S1. Properties of kaolinite and goethite.**

**Table S2. Sorption parameters in the experiment.**

**Table S3. Description of four components in microplastic leaching experiment based on the PARAFAC model.**

**Table S4. Description of three components in the two NOM based on the PARAFAC model.**

**Table S5. An Overview of the FT-ICR-MS Average Molecular Parameters for the initial DOM and post-sorption DOM solutions**

**Table S6. Adsorption kinetic parameters of six DOM on kaolinite and goethite**

**Table S7. Greenhouse gas emissions from plastics lifecycle. Data from OECD (https://doi.org/10.1787/e39547a0-en)**

**Figure S1. FTIR of microplastic (a), XRD of minerals (b), Pore volume distributions of kaolinite (c), and goethite (d)**

**Figure S2. Pictures of model soils under mineral. S: Sand; S+G: Sand + 5% goethite; S+K: Sand + 5% kaolinte**

**Figure S3. The DOM characteristics of MP-DOM during the leaching.**

**Figure S4. The four fluorescent components of the microplastic leaching experiment obtained from PARAFAC model (a) and their relative abundances (b)**

**Figure S5. The three fluorescent components in the NOM obtained from PARAFAC model (a, b, c) and their relative abundances (d)**

**Figure S6. The Zeta potential of the six DOM and two minerals.**

**Figure S7. The FTIR results of the two minerals before and after the sorption.**

**Figure S8. Excitation emission matrix (EEM) of initial DOM and post-adsorption DOM by kaolinite**

**Figure S9. The fluorescent components in the MP-DOM obtained from PARAFAC model (a, b, c), and their relative abundances of them (d).**

**Figure S10. Distributions of M/Z of molecules in DOM before and after adsorption on kaolinite and goethite.**

**Figure S11. Distributions of DBE of molecules in DOM before and after adsorption on kaolinite and goethite.**

**Figure S12. Distributions of O/C of molecules in DOM before and after adsorption on kaolinite and goethite.**

**Figure S13. Distributions of H/C of molecules in DOM before and after adsorption on kaolinite and goethite.**

**Figure S14. The changes of molecular numbers in each DOM after the sorption.**

**Figure S15. The Pearson’s correlation between DOM parameters and the generation of CO2 and MAOC.**

**Test S1 Determination of MAOC**

Briefly, 50 mL ultrapure water was added to the matrix (1:5, w: v), dispersing in 270 J mL^−1^ for 20 min using an ultrasonic generator (SN-QX-32, Shanghai, China). The suspension was passed through a 53-μm sieve to obtain MAOM ^1^. The above procedure was repeated three times for each sample. The mean recovery of minerals was 93 ± 5 %. The content of organic carbon in the mineral power was measured using an element analyzer (Flash 2000, Thermo Fisher, America), which is the mineral-associated organic carbon (MAOC).

**Text S2. DOM parameters calculation based on EEM and FT-ICR-MS.**

The index fluorescent index (FI) was defined as the ratio of emission (Em) intensities at 470 nm to those at 520 nm, with an excitation (Ex) wavelength of 370 nm, which has been widely used to differentiate between terrestrial and microbial DOM (∼1.9 for microbial sources and ∼1.4 for terrestrial sources). Humic index (HIX) was calculated as the ratio of the integral area under Em wavelength 435–480 nm to that under 300–345 + 435–480 nm at Ex wavelength 254 nm. It was used to quantify the relative degree of humification, which was increased with a stronger humic character. Biological index (BIX) was calculated as the ratio of Em wavelength at 380 nm to that at 430 nm (Ex wavelength was kept at 310 nm), and was used to decipher the presence of autochthonous biological organic matter.

For the ultrahigh-resolution mass spectra analysis, a Bruker SolariX FT-ICR-MS with a 15.0 T superconducting magnet and an ESI ion source was used. The following conditions were employed: negative ionization model, continuous infusion at a rate of 120 μL/h, an ESI needle voltage of −3.8 kV, and an ion accumulation time of 0.06 s. The detection mass range was set to m/z 100−800 Da. Peaks found in both blank samples (DI water and methyl alcohol) and DOM samples were removed. Before analysis, the instrument was calibrated with 10 mmol/L sodium formate solution. Double bond equivalence (DBE) was used for measure the number of double bonds and rings in a molecule (Eq1). Nominal oxidation state of carbon (NOSC), representing the average oxidation state of all carbons per formula independent of the chemical structure, was calculated (Eq2). Molecular lability boundary (MLB_L_) means the fraction of labile compounds (Eq3). Aromaticity index (AI*_mod_*) was calculated from the formulas to estimate the fraction of aromatic and condensed aromatic groups (Eq4). The intensity-weighted average (w) values of O/C_w_, H/C_w_, M/Z_w_, AI_w_ DBE_w_, and NOSC_w_ were calculated using Eq5, for example, O/C_w_ = O/C × X_i_, where I_i_ is the intensity of molecular formula i.

$DBE=1+\left( \frac{1}{2} \right)\left( 2C-H+N \right)$ (1)

$NOSC=4-\frac{4C+H-3N-2O-2S}{C}$ (2)

$MLB_{L}=\frac{molecules number \left( \frac{H}{C}\geq1.5 \right)}{tota molecule numbers}$ (3)

$AI_{mod}=\frac{1+C-0.5O-S-0.5H}{C-0.5O-S-N-P}$ (4)

$X_{i}=\frac{I_{i}}{\sum I_{i}}$ (5)

The DOM molecules were categorized into six groups, including (1) polycyclic aromatic formulas (AI*_mod_* > 0.66); (2) aromatic formulas (0.66 ≥ AI*_mod_* > 0.50); (3) lignin/phenolic formulas (AI*_mod_* ≤ 0.50 and H/C < 1.5), (4) nitrogen-less (N-) aliphatic compounds (2.0 > H/C ≥ 1.5 and N = 0); (5) nitrogen-containing (N+) aliphatic compounds (2.0 > H/C ≥ 1.5 and N > 0) and (6) carbohydrate-like compounds (H/C ≥ 2.0 or O/C ≥ 0.9).

**Text S3. Empirical kinetic models**

The amounts of DOC adsorbed of minerals at time t were calculated as the differences between the DOC contents of the DOM solutions at time t (1, 2, 4, 8, 16, 24, and 36 h) and those of the initial DOM solutions. Two kinetic models, Pseudo-first-order (Eq1) and Pseudo-second-order (Eq2), were adopted to depict the adsorption kinetics.

$Q_{t}=Qe (1-e^{-k_{1}t})$ (1)

$\frac{t}{Q_{t}}=\frac{1}{k_{2}\times Q_{e}^{2}}+\frac{t}{Qe}$ (2)

where Q_t_ (mg/L) and Q_e_ (mg/L) are the amounts of DOM adsorbed at time t, and at equilibrium, respectively. k_1_ (h^-1^) and k_2_ (mg g^-1^ h^-1^) are the adsorption rate constants for (Eq1) Pseudo-first-order, and (Eq2) Pseudo-second-order, respectively.

**Table S1. Properties of kaolinite and goethite.**

| Mineral | SSA *  (m^2^/g) | Pore Volume  (cm^3^/g) | Pore Size  (nm) | Cumulative pore area (m^2^/g) |
| --- | --- | --- | --- | --- |
| Kaolinite | 15.99±0.04 | 0.12±0.01 | 25.00 | 18.92±0.60 |
| Goethite | 12.27±0.11 | 0.08±0.01 | 24.06 | 12.68±0.60 |

* Specific Surface Area (SSA). The Brunauer–Emmett–Teller (BET) equation was used to calculate surface areas from the nitrogen isotherm data. Barrett-Joyner-Halenda (BJH) theory was used to obtain mesopore distributions.

**Table S2. Sorption parameters in the experiment.**

| Treatment | Initial DOC  (mg L^-1^) | Mineral mass  (mg) | Initial pH | Equilibrium DOC  (mg L^-1^) | Equilibrium pH | Lost C (w/o *) or  Absorbed C (w/) (%) |
| --- | --- | --- | --- | --- | --- | --- |
| Without Mineral | | | | | | |
| PE | 10.50±0.02 | 0 | 7.00±0.02 | 10.50±0.01 | 7.00±0.02 | 0 |
| PVC | 10.08±0.01 | 0 | 6.90±0.03 | 10.00±0.12 | 6.90±0.00 | + 0.81±2.35 |
| PLA | 10.19±0.05 | 0 | 6.92±0.00 | 10.16±0.07 | 6.93±0.01 | + 0.35±0.71 |
| PBAT | 9.71±0.03 | 0 | 6.95±0.01 | 9.79±0.28 | 6.96±0.01 | - 0.74±2.85 |
| SRNOM | 12.70±0.02 | 0 | 7.08±0.03 | 12.56±0.11 | 7.10±0.03 | + 1.04±0.86 |
| PPHA | 8.74±0.04 | 0 | 7.08±0.02 | 8.74±0.02 | 7.10±0.01 | + 0.03±0.18 |
| Kaolinite | | | | | | |
| PE | 10.50±0.02 | 15 | 7.00±0.02 | 9.02±0.02 | 7.21±0.01 | 13.97±0.31 |
| PVC | 10.08±0.01 | 15 | 6.90±0.03 | 8.68±0.04 | 7.11±0.01 | 13.86±0.81 |
| PLA | 10.19±0.05 | 15 | 6.92±0.00 | 9.62±0.17 | 7.12±0.01 | 5.62±1.67 |
| PBAT | 9.71±0.03 | 15 | 6.95±0.01 | 9.45±0.03 | 7.22±0.00 | 2.62±0.27 |
| SRNOM | 12.70±0.02 | 15 | 7.08±0.03 | 10.48±0.01 | 7.49±0.02 | 17.46±0.11 |
| PPHA | 8.74±0.04 | 15 | 7.08±0.02 | 6.31±0.13 | 7.35±0.01 | 27.85±1.49 |
| Goethite | | | | | | |
| PE | 10.50±0.02 | 15 | 7.00±0.02 | 8.12±0.06 | 6.90±0.01 | 18.72±1.23 |
| PVC | 10.08±0.01 | 15 | 6.90±0.03 | 8.26±0.02 | 6.49±0.00 | 18.05±0.40 |
| PLA | 10.19±0.05 | 15 | 6.92±0.00 | 9.38±0.03 | 6.23±0.01 | 8.02±0.34 |
| PBAT | 9.71±0.03 | 15 | 6.95±0.01 | 9.33±0.07 | 6.48±0.02 | 3.88±0.76 |
| SRNOM | 12.70±0.02 | 15 | 7.08±0.03 | 9.03±0.12 | 6.88±0.01 | 28.91±0.95 |
| PPHA | 8.74±0.04 | 15 | 7.08±0.02 | 4.68±0.05 | 6.72±0.00 | 47.02±0.56 |

* w/o: without minerals; w/: with minerals

**Table S3. Description of four components in microplastic leaching experiment based on the PARAFAC model.**

| Components | Description | References |
| --- | --- | --- |
| C1 | Terrestrial humic-like components | Lee et al., (2020)^2^ |
| C2 | Natural soil fulvic compounds | Hong et al., (2021)^3^ |
| C3 | Protein-like components | Yamashita et al., (2010) ^4^ |
| C4 | Protein-like components | (Kida et al.,) (2019) ^5^ |

**Table S4. Description of three components in the two NOM based on the PARAFAC model.**

| Components | Description | References |
| --- | --- | --- |
| NC1 | Humic-like components | Ren et al., (2021)^6^ |
| NC2 | Fulvic acid, humic-like substances | Ren et al., (2021)^6^ |
| NC3* | quinone-like components | Cory and Mcknight, (2005) ^7^ |

* The NC3 is based on the similarity of >90% obtained using OpenFluor database.

Other components are based on the similarity of >95%.

**Table S5. An Overview of the FT-ICR-MS Average Molecular Parameters for the initial DOM and post-sorption DOM solutions**

| Treatment | Formula number | M/Z_w_ | O/C_w_ | H/C_w_ | AI_w_ | DBE_w_ | NOSC_w_ | MLB_L_ |
| --- | --- | --- | --- | --- | --- | --- | --- | --- |
| Conventional microplastic-derived DOM | | | | | | | | |
| PE | 2279±53 | 339±5 | 0.29±0.02 | 1.59±0.05 | 0.10±0.00 | 5.18±0.09 | -0.87±0.08 | 0.50±0.07 |
| K-PE | 1959±102 | 366±8 | 0.27±0.06 | 1.57±0.10 | 0.11±0.00 | 5.76±0.08 | -0.89±0.06 | 0.43±0.06 |
| G-PE | 1476±112 | 357±4 | 0.31±0.04 | 1.55±0.12 | 0.11±0.01 | 5.82±0.12 | -0.78±0.07 | 0.49±0.08 |
| PVC | 3201±201 | 347±7 | 0.32±0.06 | 1.59±0.06 | 0.07±0.00 | 4.95±0.21 | -0.82±0.08 | 0.48±0.12 |
| K-PVC | 1703±63 | 375±3 | 0.25±0.03 | 1.67±0.23 | 0.08±0.00 | 4.82±0.08 | -1.06±0.12 | 0.47±0.08 |
| G-PVC | 2161±35 | 402±5 | 0.30±0.01 | 1.60±0.06 | 0.08±0.00 | 5.59±0.08 | -0.86±0.21 | 0.51±0.03 |
| Biodegradable microplastic-derived DOM | | | | | | | | |
| PLA | 920±35 | 488±4 | 0.55±0.03 | 1.53±0.05 | 0.001±0.000 | 6.41±0.07 | -0.42±0.09 | 0.29±0.05 |
| K-PLA | 433±64 | 404±3 | 0.55±0.03 | 1.50±0.08 | 0.007±0.000 | 6.57±0.07 | -0.35±0.08 | 0.44±0.02 |
| G-PLA | 458±75 | 382±6 | 0.54±0.02 | 1.55±0.07 | 0.003±0.000 | 5.61±0.02 | -0.45±0.05 | 0.53±0.08 |
| PBAT | 1124±35 | 414±7 | 0.53±0.05 | 1.60±0.10 | -0.03±0.00 | 5.07±0.14 | -0.52±0.06 | 0.60±0.06 |
| K-PBAT | 675±62 | 371±10 | 0.52±0.04 | 1.59±0.06 | -0.01±0.01 | 4.82±0.15 | -0.52±0.06 | 0.52±0.03 |
| G-PBAT | 692±28 | 366±12 | 0.54±0.04 | 1.62±0.05 | -0.03±0.01 | 4.24±0.08 | -0.52±0.14 | 0.52±0.00 |
| Natural dissolved organic carbon (NOM) | | | | | | | | |
| SRNOM | 4132±103 | 472±13 | 0.53±0.04 | 1.05±0.05 | 0.35±0.02 | 11.62±0.62 | 0.02±0.00 | 0.04±0.00 |
| K-SRMOM | 3451±201 | 450±5 | 0.51±0.03 | 1.10±0.07 | 0.33±0.01 | 10.72±0.24 | -0.06±0.01 | 0.04±0.00 |
| G-SRMOM | 3526±231 | 441±7 | 0.49±0.01 | 1.13±0.06 | 0.32±0.00 | 10.25±0.32 | -0.13±0.00 | 0.04±0.01 |
| HA | 4695±268 | 402±18 | 0.45±0.03 | 0.96±0.10 | 0.45±0.03 | 11.74±0.14 | 0.06±0.00 | 0.11±0.01 |
| K-HA | 4430±402 | 400±21 | 0.44±0.04 | 1.07±0.06 | 0.38±0.01 | 10.32±0.15 | -0.08±0.00 | 0.10±0.00 |
| G-HA | 3441±105 | 390±10 | 0.40±0.06 | 1.35±0.03 | 0.21±0.02 | 7.39±0.06 | -0.42±0.03 | 0.13±0.00 |

K-XX: DOM post-adsorption with kaolinite

G-XX: DOM post-adsorption with goethite

**Table S6. Adsorption kinetic parameters of six DOM on kaolinite and goethite**

| Treatment | Q_e_ (mg kg^-1^) | k_1_ (h^-1^)  k_2_ (mg g^-1^ h^-1^) | R^2^ | Treatment | Q_e_ | k_1_ (h^-1^)  k_2_ (mg g^-1^ h^-1^) | R^2^ |
| --- | --- | --- | --- | --- | --- | --- | --- |
| Pseudo-first-order | | | | | | | |
| K-PE | 0.71±0.03 | 0.89±0.17 | 0.74 | G-PE | 0.95±0.02 | 1.96±0.39 | 0.50 |
| K-PVC | 0.70±0.02 | 0.80±0.13 | 0.83 | G-PVC | 0.89±0.01 | 1.79±0.24 | 0.74 |
| K-PLA | 0.54±0.03 | 0.52±0.12 | 0.88 | G-PLA | 0.78±0.02 | 0.81±0.10 | 0.93 |
| K-PBAT | 0.24±0.01 | 0.84±0.20 | 0.80 | G-PBAT | 0.37±0.00 | 1.14±0.07 | 0.97 |
| K-SRNOM | 2.19±0.05 | 1.80±0.31 | 0.61 | G-SRNOM | 3.03±0.03 | 0.87±0.05 | 0.98 |
| K-HA | 1.42±0.14 | 0.21±0.07 | 0.76 | G-HA | 3.96±0.18 | 0.74±0.16 | 0.74 |
| Pseudo-second-order | | | | | | | |
| K-PE | 0.75±0.02 | 2.08±0.45 | 0.91 | G-PE | 0.98±0.01 | 4.89±1.06 | 0.86 |
| K-PVC | 0.73±0.03 | 1.98±0.52 | 0.87 | G-PVC | 0.91±0.01 | 4.95±0.58 | 0.95 |
| K-PLA | 0.60±0.05 | 1.18±0.52 | 0.83 | G-PLA | 0.83±0.04 | 1.63±0.59 | 0.82 |
| K-PBAT | 0.26±0.02 | 5.39±2.96 | 0.67 | G-PBAT | 0.38±0.01 | 5.80±0.90 | 0.94 |
| K-SRNOM | 2.26±0.03 | 1.92±0.34 | 0.90 | G-SRNOM | 3.20±0.06 | 0.48±0.07 | 0.96 |
| K-HA | 1.59±0.17 | 0.19±0.09 | 0.83 | G-HA | 4.21±0.14 | 0.29±0.07 | 0.90 |

K-XX: adsorption kinetic model parameters of kaolinite

G-XX: adsorption kinetic model parameters of goethite

k_1_ (h^-1^) and k_2_ (mg g^-1^ h^-1^) are the adsorption rate constants for Pseudo-first-order, and Pseudo-second-order, respectively.

**Table S7. Greenhouse gas emissions from plastics lifecycle. Data from OECD (https://doi.org/10.1787/e39547a0-en)**

|  | 2019 | 2020 | 2021 | 2022 | 2023 | 2324 | 2025 | 2026 | 2027 | 2028 | 2029 | 2030 | 2031 | 2032 | 2033 | 2034 |
| --- | --- | --- | --- | --- | --- | --- | --- | --- | --- | --- | --- | --- | --- | --- | --- | --- |
| Plastic Production & Conversion  Plastic End-of-life | 1605.43  192.86 | 1530.51  192.13 | 1570.11  196.15 | 1624.15  201.25 | 1663.48  206.02 | 1701.38  210.23 | 1740.92  214.39 | 1783.46  218.84 | 1824.5  223.43 | 1869.18  228.31 | 1915.88  233.35 | 1964.39  238.47 | 2012.23  243.67 | 2061.53  249.01 | 2110.9  254.51 | 2163.1  260.22 |

|  | 2035 | 2036 | 2037 | 2038 | 2039 | 2040 | 2041 | 2042 | 2043 | 2044 | 2045 | 2046 | 2047 | 2048 | 2049 | 2050 |
| --- | --- | --- | --- | --- | --- | --- | --- | --- | --- | --- | --- | --- | --- | --- | --- | --- |
| Plastic Production & Conversion  Plastic End-of-life | 2214.47  266.09 | 2265.04  272.04 | 2317.4  278.26 | 2371.08  284.65 | 2427.1  291.25 | 2483.91  298.02 | 2540.71  304.92 | 2598.25  311.99 | 2659.72  319.23 | 2724.22  326.76 | 2788.62  334.44 | 2854.86  342.3 | 2920.35  350.3 | 2986.96  358.44 | 3054.65  366.75 | 3124.17  375.25 |

|  | 2051 | 2052 | 2053 | 5054 | 2055 | 2056 | 2057 | 2058 | 2059 | 2060 |
| --- | --- | --- | --- | --- | --- | --- | --- | --- | --- | --- |
| Plastic Production & Conversion  Plastic End-of-life | 3191.97  384.01 | 3260.85  393.18 | 3329.6  402.45 | 3398.61  412.07 | 3466.59  421.85 | 3534.22  432.06 | 3601.02  442.43 | 3668.39  442.43 | 3734.39  464.27 | 3802.79  475.9 |


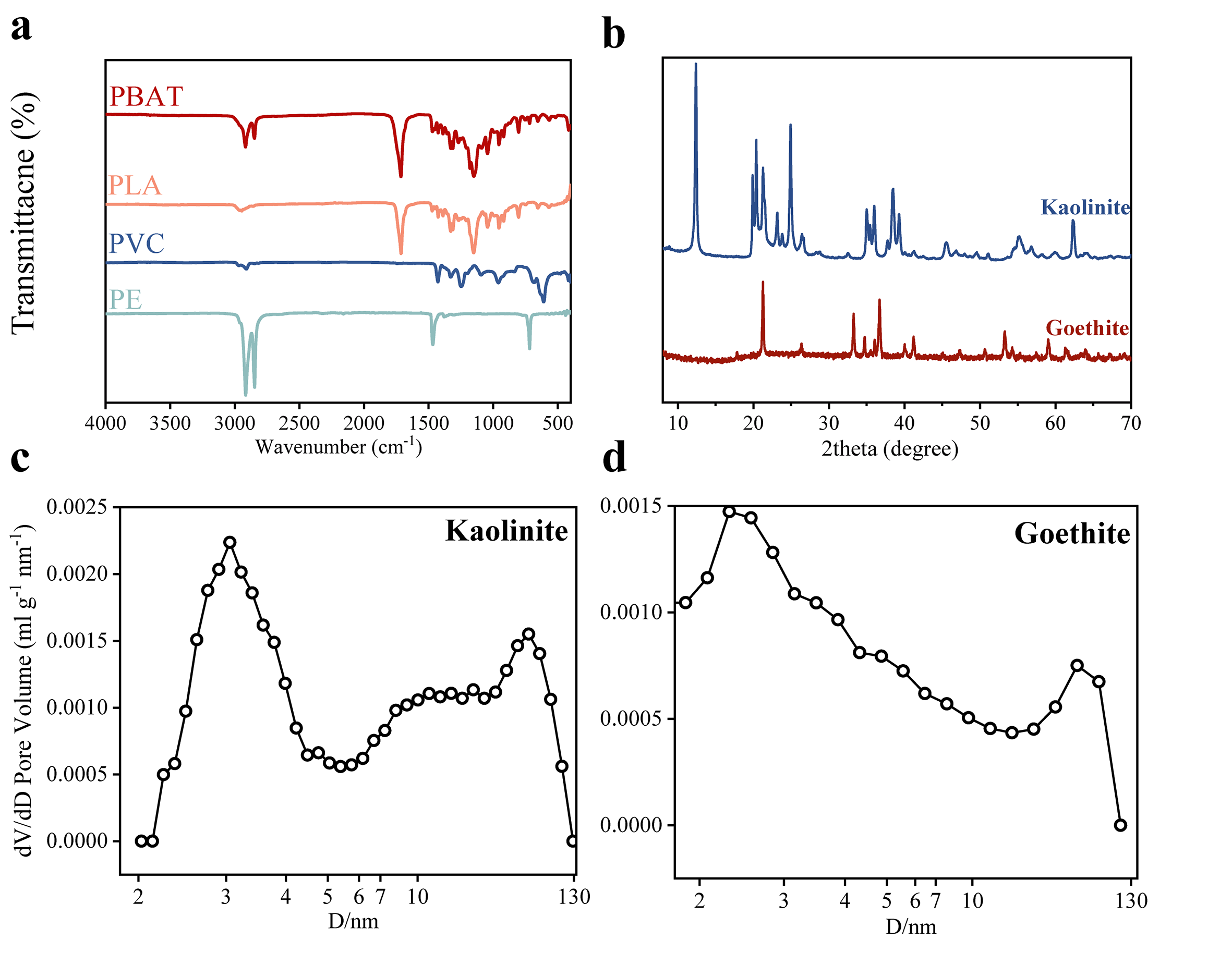


**Figure S1. FTIR of microplastic (a), XRD of minerals (b), Pore volume distributions of kaolinite (c), and goethite (d)**


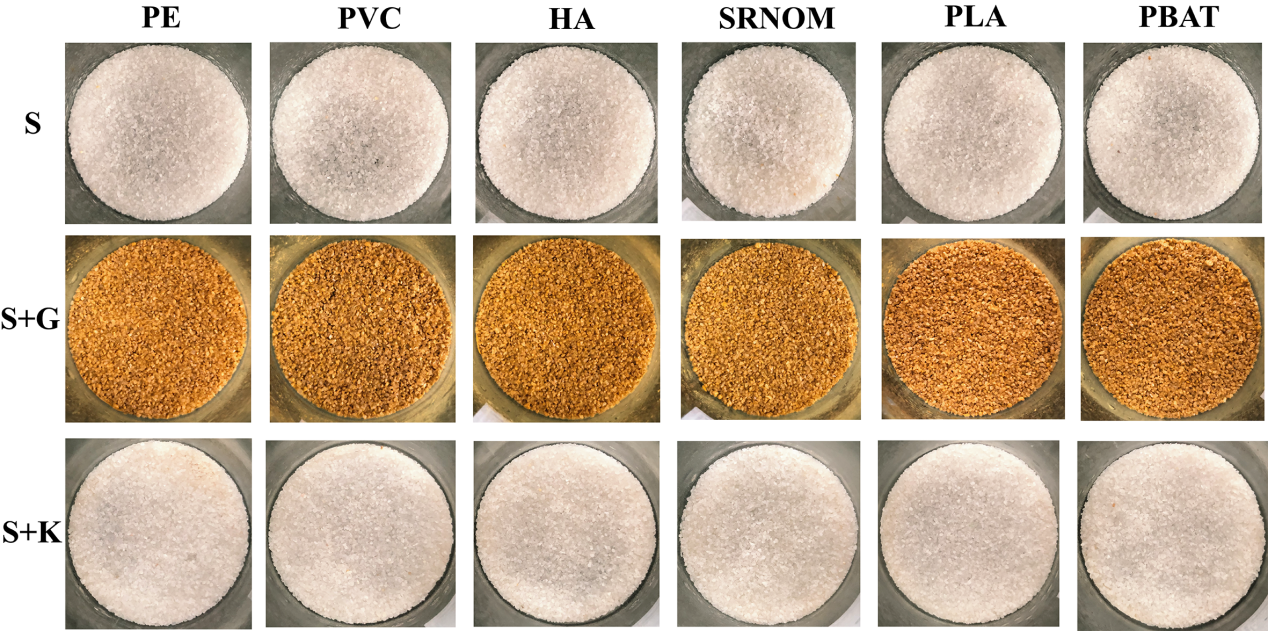


**Figure S2. Pictures of model soils under mineral. S: Sand; S+G: Sand + 5% goethite; S+K: Sand + 5% kaolinte**


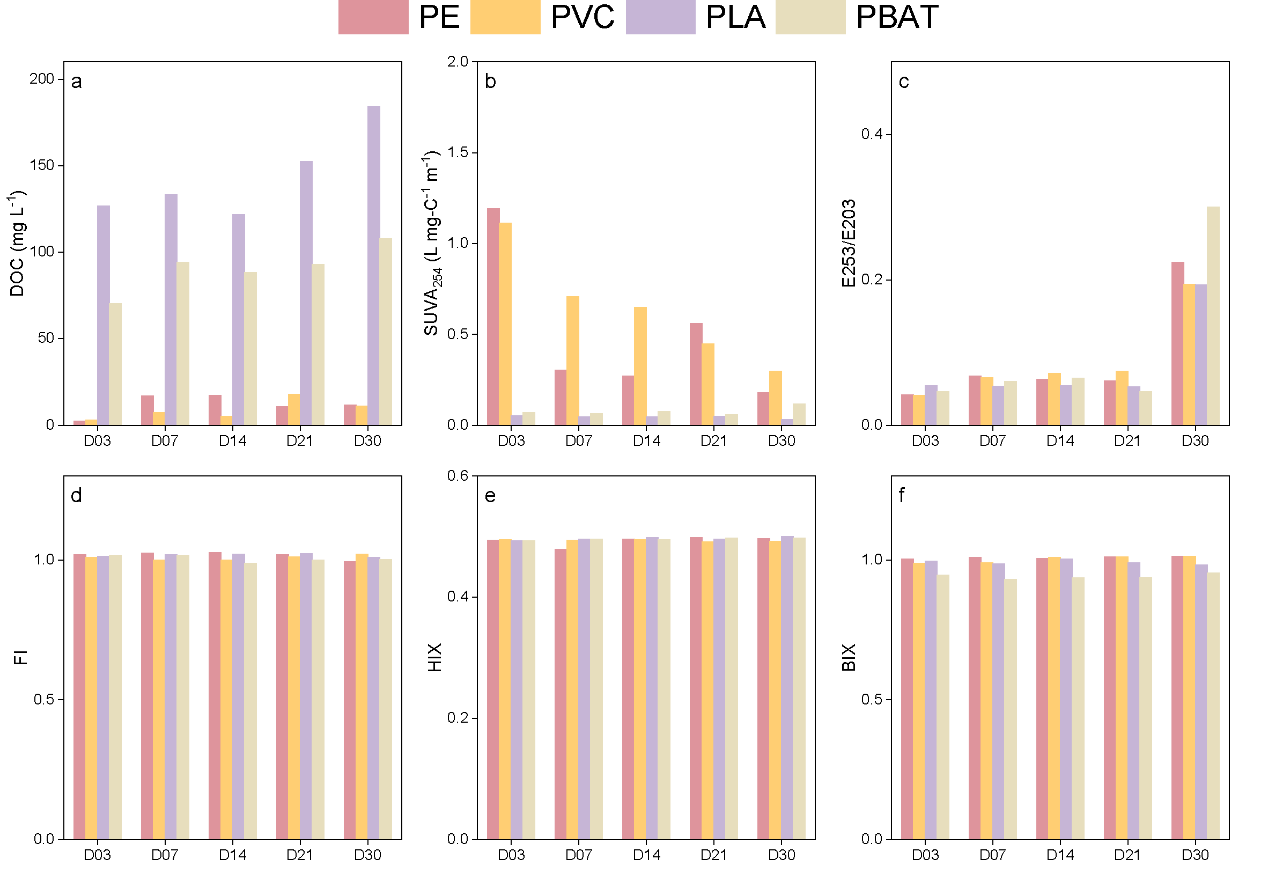


**Figure S3. The DOM characteristics of MP-DOM during the leaching.**

**
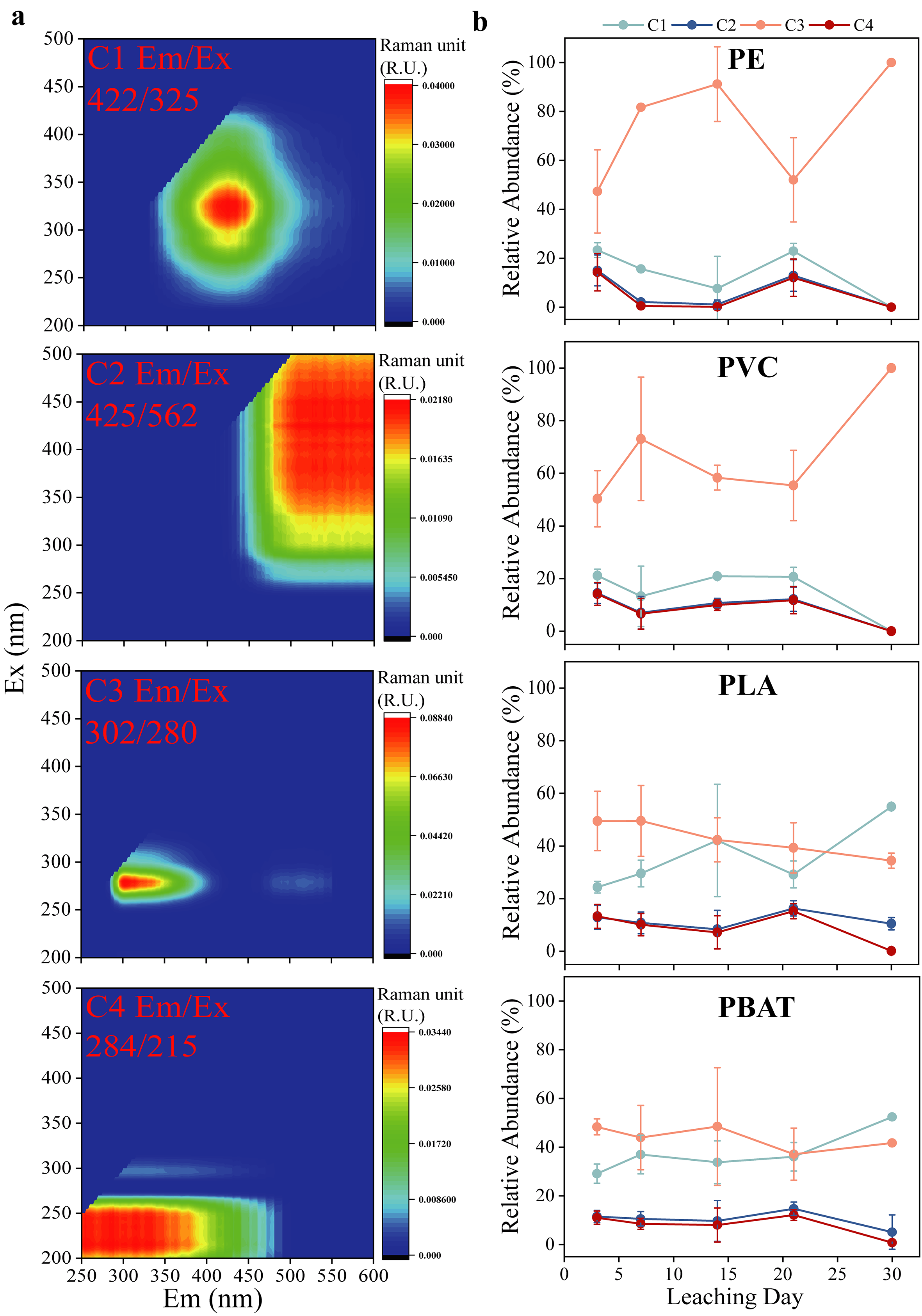
**

**Figure S4. The four fluorescent components of the microplastic leaching experiment obtained from PARAFAC model (a) and their relative abundances (b)**

**
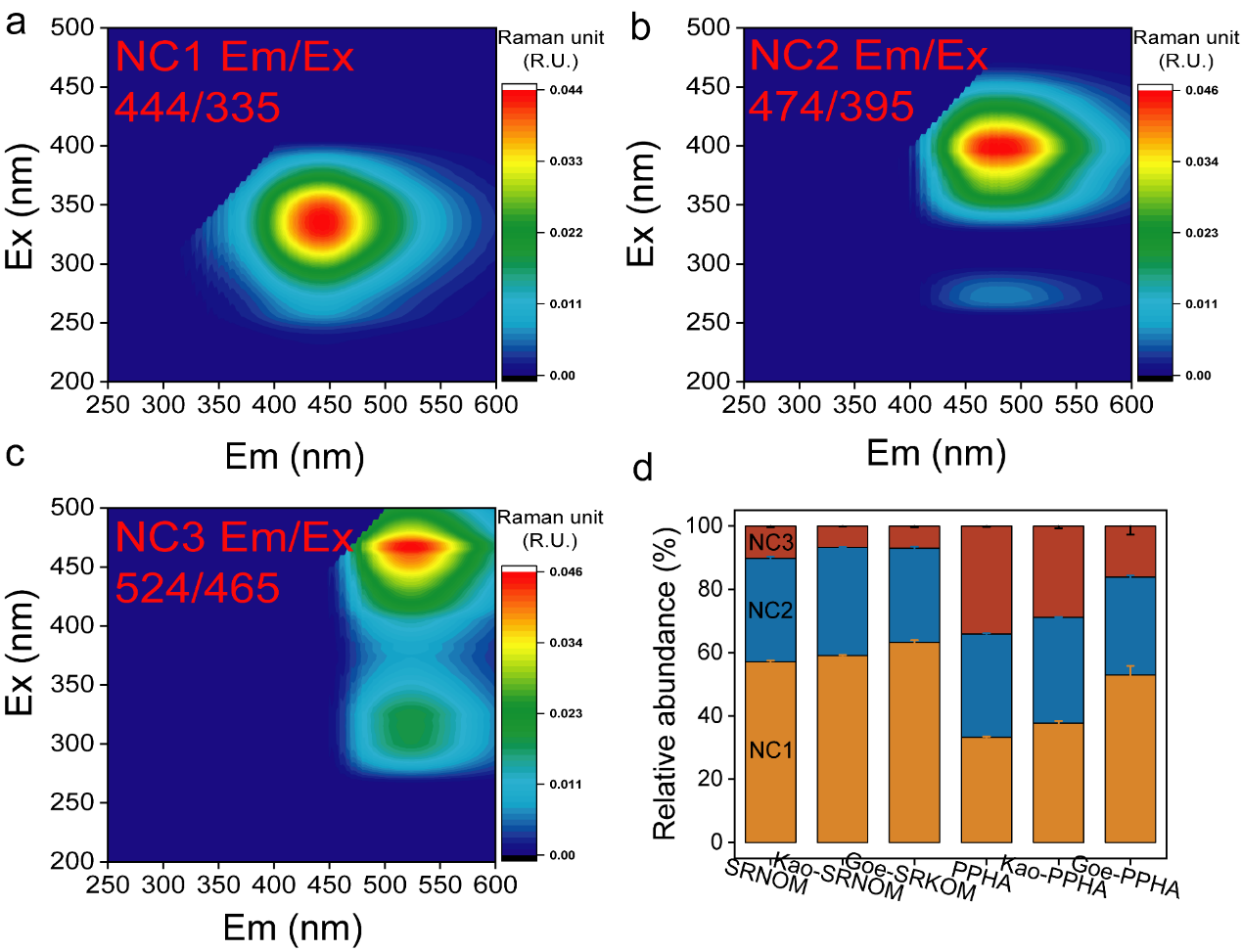
**

**Figure S5. The three fluorescent components in the NOM obtained from PARAFAC model (a, b, c) and their relative abundances (d). SRNOM: the initial SRNOM; K-SRNOM: post-adsorption SRNOM solutions by kaolinite; G-SRNOM: post-adsorption SRNOM solutions by goethite**

**Figure S6. The Zeta potential of the six DOM and two minerals.**


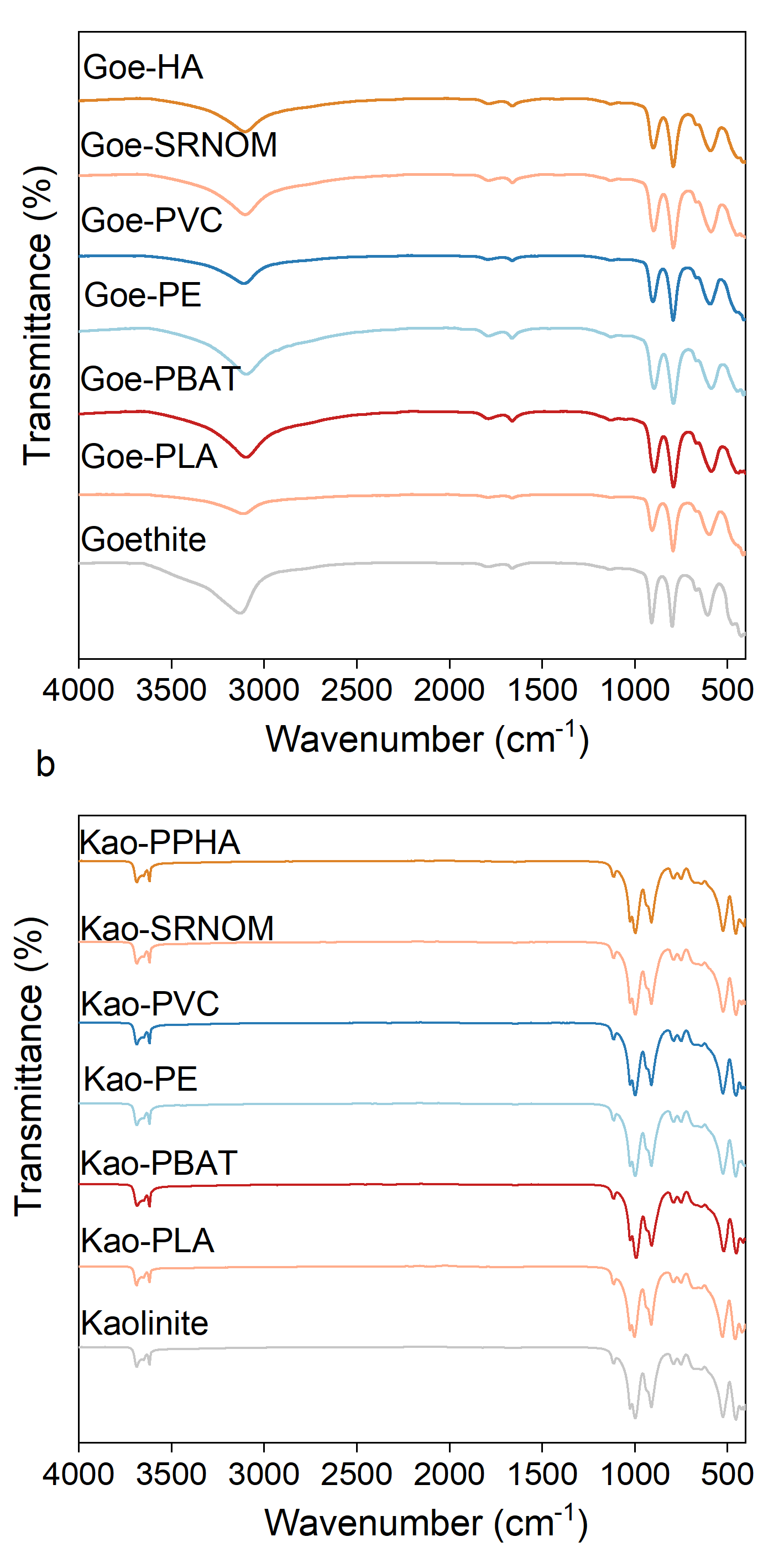


**Figure S7. The FTIR results of the two minerals before and after the sorption.**

**
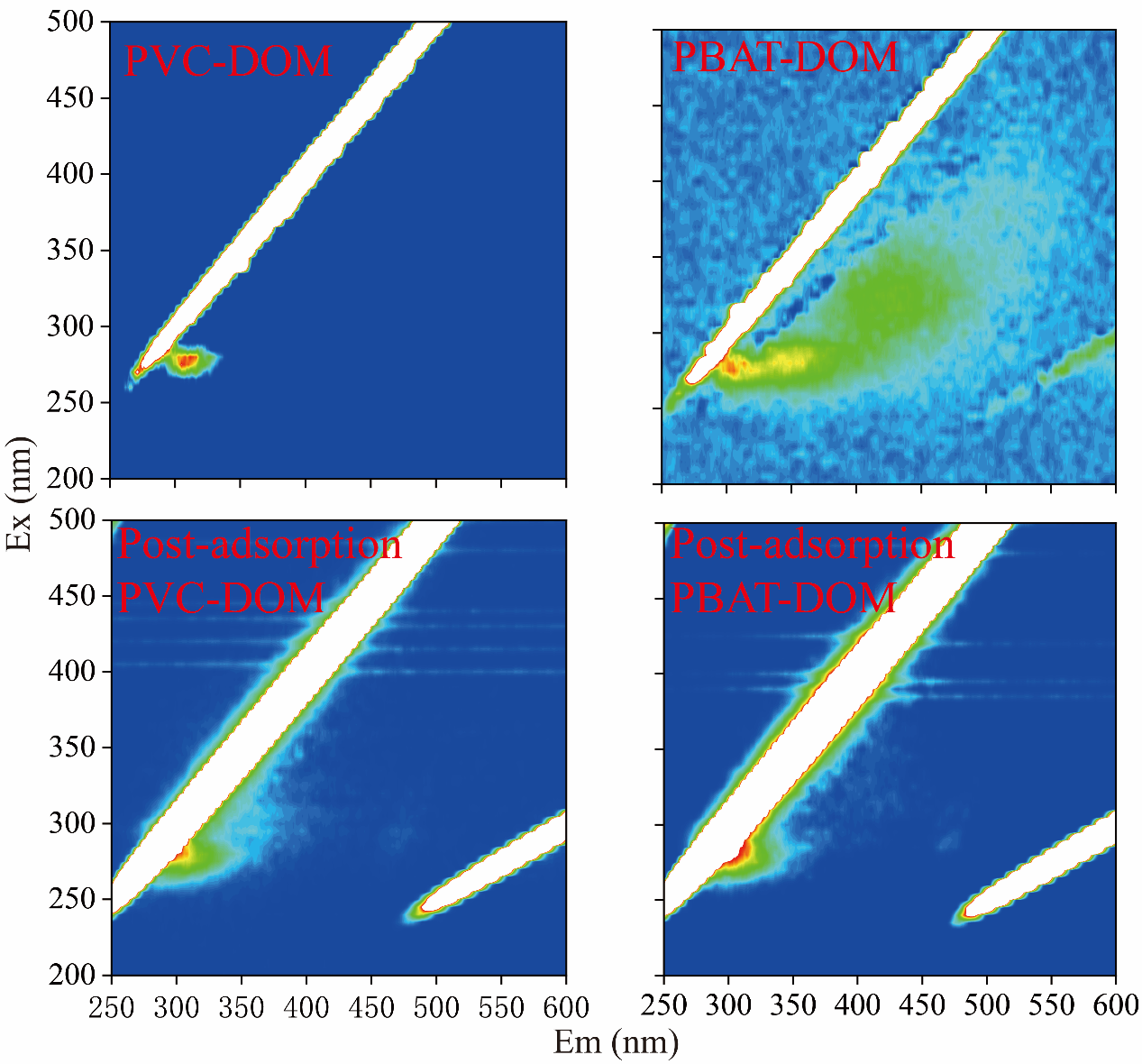
**

**Figure S8. Excitation emission matrix (EEM) of initial DOM and post-adsorption DOM by kaolinite**


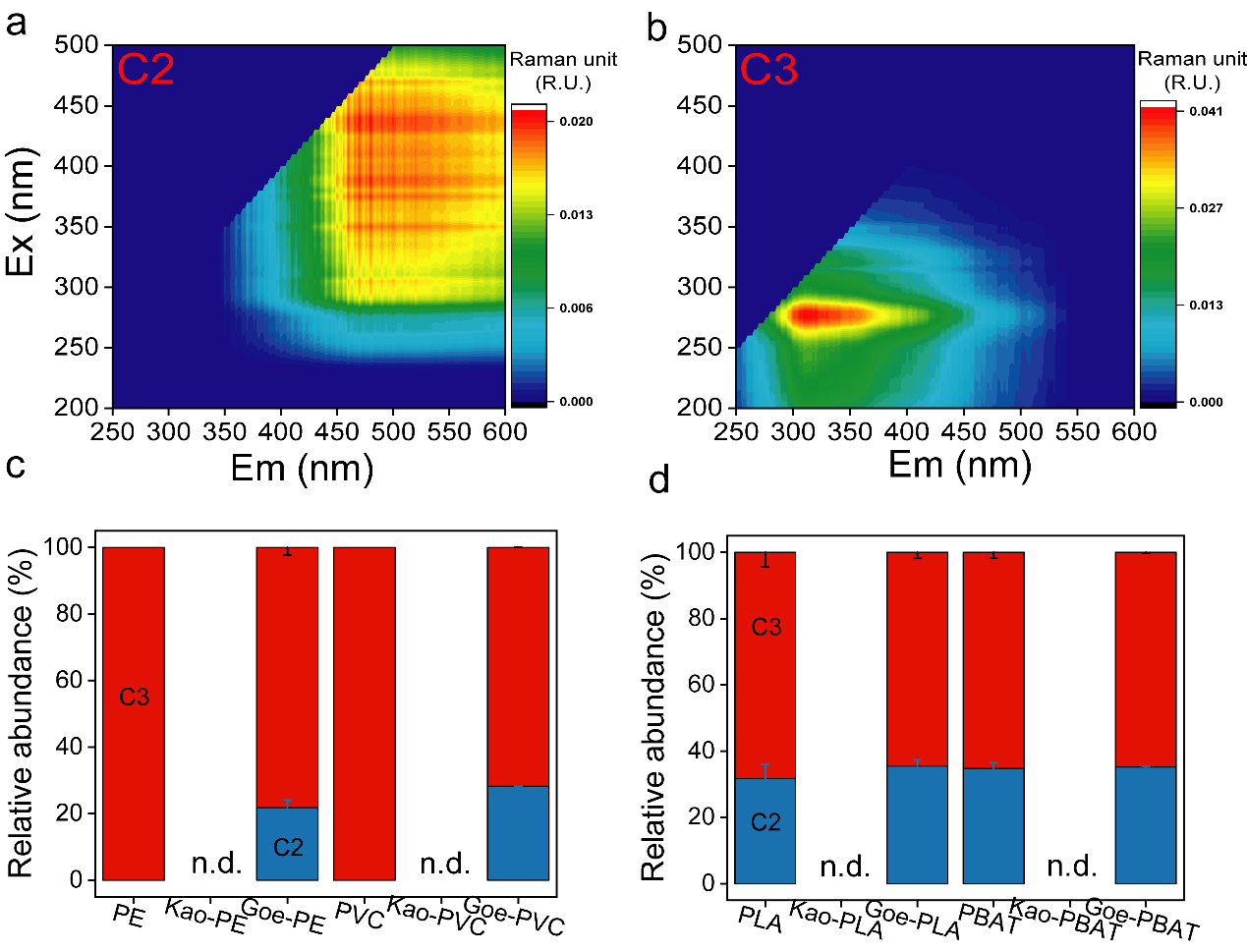


**Figure S9. The fluorescent components in the MP-DOM obtained from PARAFAC model (a, b, c), and their relative abundances of them (d). For example, PE: the initial PE-DOM; Kao-PE: post-adsorption PE-DOM solutions by kaolinite; Goe-PE: post-adsorption PE-DOM solutions by goethite.**

**Figure S10. Distributions of M/Z of molecules in DOM before and after adsorption on kaolinite and goethite.**

**Figure S11. Distributions of DBE of molecules in DOM before and after adsorption on kaolinite and goethite.**

**Figure S12. Distributions of O/C of molecules in DOM before and after adsorption on kaolinite and goethite.**

**Figure S13. Distributions of H/C of molecules in DOM before and after adsorption on kaolinite and goethite.**


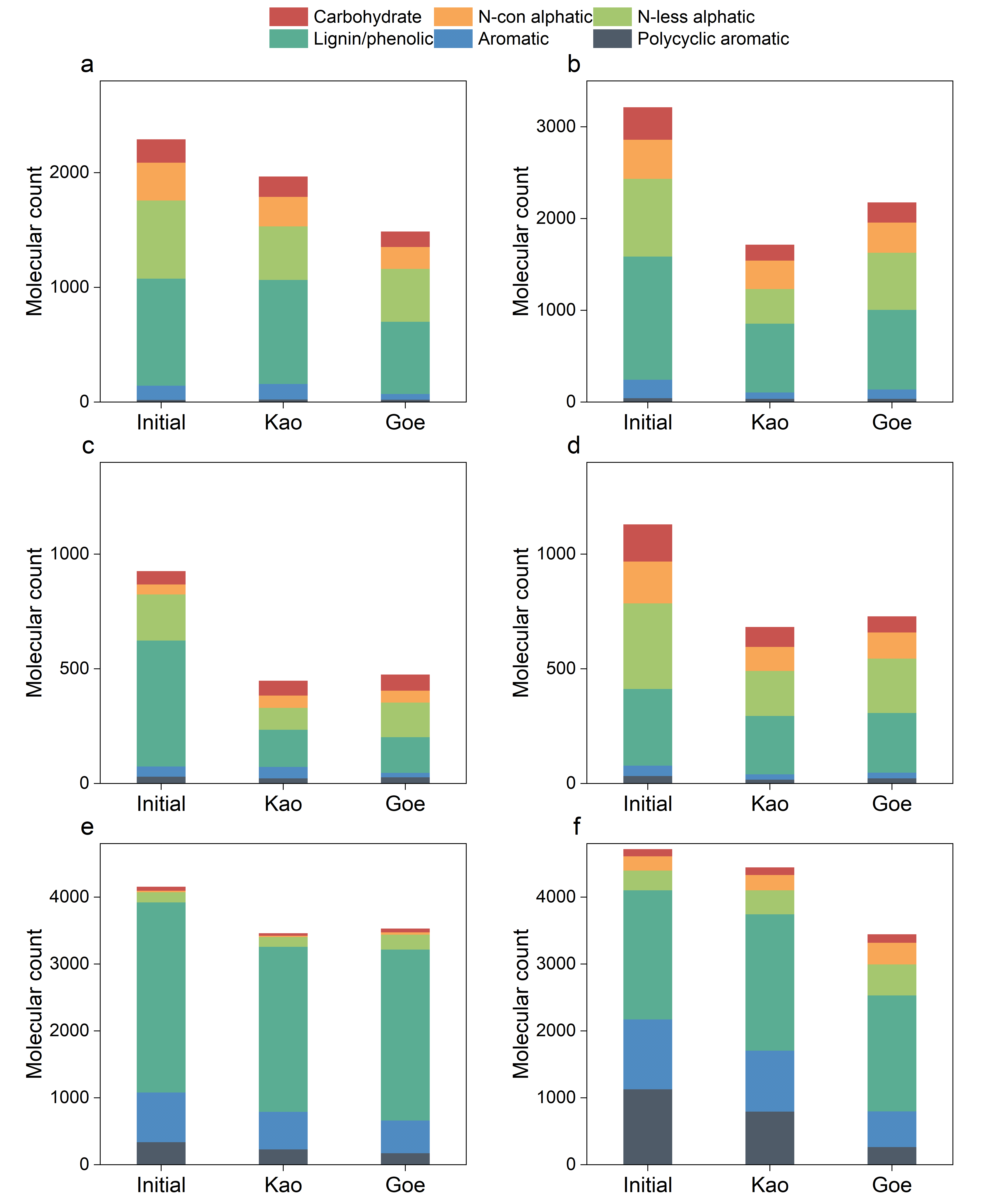


**Figure S14. The changes of molecular numbers in each DOM after the sorption.**


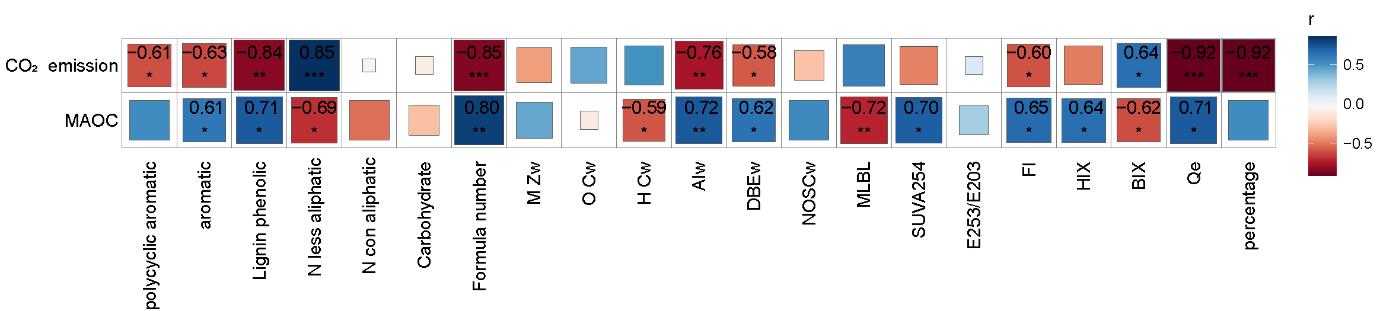


**Figure S15. The Pearson’s correlation between DOM parameters and the generation of CO_2_ and MAOC. DOM parameters include the relative abundances of the six subcategories (polycyclic aromatic-like, aromatic-like, lignin/phenolic-like, N-less aliphatic-like, N-con aliphatic-like, and carbohydrate-like), formula number, molecular weight (M/Z, intensity-weighted), oxygen carbon ratio (O/C, intensity-weighted), hydrogen carbon ratio (H/C, intensity-weighted), modified aromaticity index (AI, intensity-weighted), double bond equivalence (DBE, intensity-weighted), nominal oxidation state of carbon (NOSC, intensity-weighted), percentage of labile-like compounds (MLBL), specific ultraviolet absorbance at 254 nm (SUVA_254_), ratio of absorbance at 253 nm to 203 nm (E253/E203), fluorescent index (FI), humic index (HIX), biological index (BIX), the equilibrium concentration of DOM on minerals (Qe), and the sorbed percentage of DOM on minerals (sorption)**

**References:**

(1) Zou, Z.; Ma, L.; Wang, X.; Chen, R.; Jones, D.L.; Bol, R.; Wu, D.; Du, Z. Decadal application of mineral fertilizers alters the molecular composition and origins of organic matter in particulate and mineral-associated fractions. *Soil Biology and Biochemistry*. **2023**, *182*, 109042.

(2) Lee, S.; Kim, T.; Kim, G. Tracing terrestrial versus marine sources of dissolved organic carbon in a coastal bay using stable carbon isotopes. *Biogeosciences*. **2020**, *17*(1), 135-144.

(3) Hong, H.; Wu, S.; Wang, Q.; Dai, M.; Qian, L.; Zhu, H.; Li, J.; Zhang, J.; Liu, J.; Li, J.; Lu, H.; Yan, C. Fluorescent dissolved organic matter facilitates the phytoavailability of copper in the coastal wetlands influenced by artificial topography. *Sci. Total Environ.* **2021**, *790*, 147855.

(4) Yamashita, Y.; Cory, R.M.; Nishioka, J.; Kuma, K.; Tanoue, E.; Jaffé, R. Fluorescence characteristics of dissolved organic matter in the deep waters of the Okhotsk Sea and the northwestern North Pacific Ocean. *Deep Sea Research Part II: Topical Studies in Oceanography*. **2010**, *57*(16), 1478-1485.

(5) Kida, M.; Kojima, T.; Tanabe, Y.; Hayashi, K.; Kudoh, S.; Maie, N.; Fujitake, N. Origin, distributions, and environmental significance of ubiquitous humic-like fluorophores in Antarctic lakes and streams. *Water Res.* **2019**, *163*, 114901.

(6) Ren, W.; Wu, X.; Ge, X.; Lin, G.; Zhou, M.; Long, Z.; Yu, X.; Tian, W. Characteristics of dissolved organic matter in lakes with different eutrophic levels in southeastern Hubei Province, China. *J. Oceanol. Limnol.* **2021**, *39*(4), 1256-1276.

(7) Cory, R.M.; McKnight, D.M. Fluorescence Spectroscopy Reveals Ubiquitous Presence of Oxidized and Reduced Quinones in Dissolved Organic Matter. *Environ. Sci. Technol.* **2005**, *39*(21), 8142-8149.

(8) Lee, Y.K.; Murphy, K.R.; Hur, J. Fluorescence Signatures of Dissolved Organic Matter Leached from Microplastics: Polymers and Additives. *Environ. Sci. Technol.* **2020**, *54*(19), 11905-11914.
